# The 3D Bioprinted Scaffolds for Wound Healing

**DOI:** 10.3390/pharmaceutics14020464

**Published:** 2022-02-21

**Authors:** Pablo Edmundo Antezana, Sofia Municoy, María Inés Álvarez-Echazú, Pablo Luis Santo-Orihuela, Paolo Nicolás Catalano, Taleb H. Al-Tel, Firoz Babu Kadumudi, Alireza Dolatshahi-Pirouz, Gorka Orive, Martin Federico Desimone

**Affiliations:** 1 Facultad de Farmacia y Bioquímica, Instituto de Química y Metabolismo del Fármaco (IQUIMEFA), Universidad de Buenos Aires, Consejo Nacional de Investigaciones Científicas y Técnicas (CONICET), Junín 956, Buenos Aires 1113, Argentina; pablo.e.antezana@gmail.com (P.E.A.); smunicoy@gmail.com (S.M.); inesalvarezechazu@gmail.com (M.I.Á.-E.); psorihuela@gmail.com (P.L.S.-O.); paoloncatalano@gmail.com (P.N.C.); 2 Centro de Investigaciones en Plagas e Insecticidas (CIPEIN), Instituto de Investigaciones Científicas y Técnicas para la Defensa CITEDEF/UNIDEF, Consejo Nacional de Investigaciones Científicas y Técnicas, Buenos Aires, Argentina (CONICET), Juan B. de La Salle 4397, Villa Martelli, Buenos Aires 1603, Argentina; 3 Departamento de Micro y Nanotecnología, Instituto de Nanociencia y Nanotecnología, CNEA-CONICET, Av. General Paz 1499, San Martín 1650, Argentina; 4 Sharjah Institute for Medical Research and College of Pharmacy, University of Sharjah, Sharjah P.O. Box 27272, United Arab Emirates; taltal@sharjah.ac.ae; 5 Department of Health Technology, Technical University of Denmark, 2800 Kongens Lyngby, Denmark; firozbabu23@gmail.com (F.B.K.); alirezadolatshahipirouz@gmail.com (A.D.-P.); 6 Laboratory of Pharmaceutics, NanoBioCel Group, School of Pharmacy, University of the Basque Country UPV/EHU, Paseo de la Universidad 7, 01006 Vitoria-Gasteiz, Spain; gorka.orive@ehu.eus; 7 Biomedical Research Networking Centre in Bioengineering, Biomaterials and Nanomedicine (CIBER-BBN), 01006 Vitoria-Gasteiz, Spain; 8 Bioaraba, NanoBioCel Research Group, 01006 Vitoria-Gasteiz, Spain; 9 University Institute for Regenerative Medicine and Oral Implantology-UIRMI (UPV/EHU-Fundación Eduardo Anitua), 01007 Vitoria-Gasteiz, Spain; 10 Singapore Eye Research Institute, The Academia, 20 College Road, Discovery Tower, Singapore 169856, Singapore

**Keywords:** tissue engineering, three-dimensional bioprinted scaffolds, wound healing, three-dimensional printing technology, bioinks

## Abstract

Skin tissue engineering and regeneration aim at repairing defective skin injuries and progress in wound healing. Until now, even though several developments are made in this field, it is still challenging to face the complexity of the tissue with current methods of fabrication. In this review, short, state-of-the-art on developments made in skin tissue engineering using 3D bioprinting as a new tool are described. The current bioprinting methods and a summary of bioink formulations, parameters, and properties are discussed. Finally, a representative number of examples and advances made in the field together with limitations and future needs are provided.

## 1. Introduction

Tissue engineering has become an important research area in the past two decades since it allows restoration of the functionality of damaged tissues and organs [1]. The skin is the outer covering and the largest organ of the human body. Skin tissue engineering and regeneration has been favourable for making important advances in wound healing by designing constructs with similar structures and biological functions of native tissues [2]. With the advent of 3D printing technology, much effort has been invested to transform conventional approaches and develop new 3D bioprinting techniques that can produce more complex, functional, and personalised three-dimensional architectures with better imitation behaviour [3,4]. In this context, it is important to analyse the different types of 3D printing technologies and their associated printing parameters [5,6,7].

The 3D bioprinting of tissues is an additive manufacturing technique used to create biocompatible 3D structures mimicking the natural systems through a computer-made design. Unlike the traditional skin regeneration methods, 3D bioprinted dermal replacements are superior in the automation and normalisation for clinical uses and accuracy in the incorporation of living cells, growth factors, and other biomolecules.

The fabrication of the three-dimensional complex matrices for wound healing and skin engineering by 3D bioprinting requires the use of bioprintable materials known as bioinks [8,9]. A wide range of natural polymer hydrogels have been used as bioinks, including collagen, alginate, chitosan, hyaluronic acid, and cellulose [10]. Synthetic-based biopolymers have also been applied as printable materials to improve mechanics of the 3D constructs [11,12]. Regardless of the origin of these bioinks, they should possess some crucial properties, such as good printability, mechanical stability, biocompatibility, biodegradability, non-toxicity, high availability, and high shape fidelity, after the printing processes. Furthermore, the selection and source of living cells are high-priority factors when designing the bioink as they have a straight impact on the immune response after the implantation of the printed scaffolds. In this sense, primary skin cells (fibroblasts, keratinocytes, and melanocytes) are preferred for co-culturing during skin bioprinting constructs. It is important that the printed tissue/organ constantly ensures the normal cellular activities, including cell migration capacity and proliferation rate [13]. Indeed, the viscoelastic behaviour of bioinks influences their printability, but also the ability of cells to remodel the matrix, spread, migrate, and proliferate [14].

Nowadays, skin bioprinting has gained popularity and big companies are investing in this area. As example of this, L’Oreal USA has signed a Research Collaboration Agreement with a 3D bioprinting company in order to produce skin models for testing cosmetic products. In addition, Rokit, an important leader in 3D printers, is collaborating with a Singapore government project in order to bioprint human skin tissue [15].

According to UnivDatos Market Insights [16], the worldwide 3D bioprinting market of living human tissues/organs is likely to achieve a market value of USD 2846.3 million by 2027 from USD 651.6 million in 2019. This market is segmented as research and clinical according to the application of the final product. The clinical applications market consists of skin, bone, cartilage, and blood vessel printing, among others. Particularly, the use of the 3D bioprinting approach for wound healing and skin regeneration started in 2009 [17], reaching several published studies around 19 and 70 in 2019 and 2020, respectively. Markets reported that skin bioprinting is expected to reach a compound annual growth rate (CAGR) of 19.8% for the period of 2019–2024 [18], as a result of technological advancements in 3D bioprinters and biomaterials, and the increased demand and growing use of 3D bioprinting in pharmaceutics, cosmetics, and reconstruction and transplantation surgeries.

The advantage of 3D printing technology in the fabrication of skin scaffolds allows interconnected macro/microporosity, the use of different bioinks, and a precise geometric configuration that matches with the tissue defect. These characteristics made this technology an interesting and promising approach for the scaffold’s development [19]. In this sense, in this review we will describe different 3D printing technologies, with their associated parameters, commonly used bioinks, and the applications developed so far.

## 2. The 3D Printing Technology

**Figure 1:**
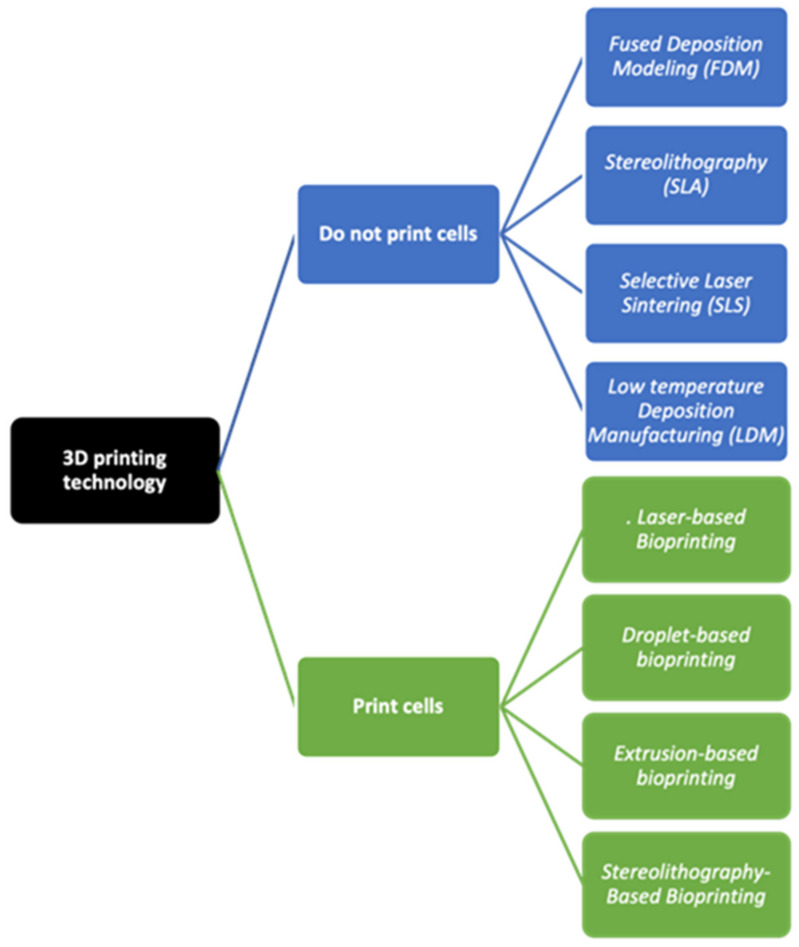
The 3D bioprinting classification.

The 3D printing technology allows the manufacturing of custom-made 3D structures with high resolution and controlled internal micro-architecture. There are different 3D printing techniques that could be used to produce scaffolds for tissue regeneration, each one with advantages and drawbacks. The 3D bioprinting is a manufacturing technique that could be used to produce artificial scaffolds or tissue constructs using a layer-by-layer deposition process. In association with tissue engineering applications, the 3D bioprinting techniques could be divided into two major groups: the ones that could not print living cells directly into the structure and the ones that can. Among the first ones, it is possible to find fused deposition modelling (FDM), stereolithography (SLA), selective laser sintering (SLS) and low-temperature deposition manufacturing (LDM). On the other hand, there are cellular bioprinting techniques that uses bioinks with viable cells in order to form the construct. These bioprinting techniques could be classified into four categories: laser-based, droplet-based, extrusion-based, and stereolithography-based bioprinting [20,21] (Figure 1).

### 2.1. Fused Deposition Modelling (FDM)

FDM 3D printing is a widely used technique in the industry due to its safety, operational efficiency, durability, and simpler, less expensive equipment [22,23]. In addition, highly reproducible and bioresorbable 3D scaffolds can be fabricated using this technique [24]. Moreover, this technique could be used to print 3D structural support for cell-laden soft materials in the printed constructs [25]. The fabrication of porous, 3D-printed chitosan scaffolds for skin tissue regeneration was achieved, showing superior healing compared to commercial patches and spontaneous healing [26]. In this vein, the use of keratinocytes, melanocytes, and fibroblast from skin donors leads to a three-dimensional pigmented human skin construct using a two-step print process using collagen [27].

#### 2.1.1. FDM Process

**Figure 2:**
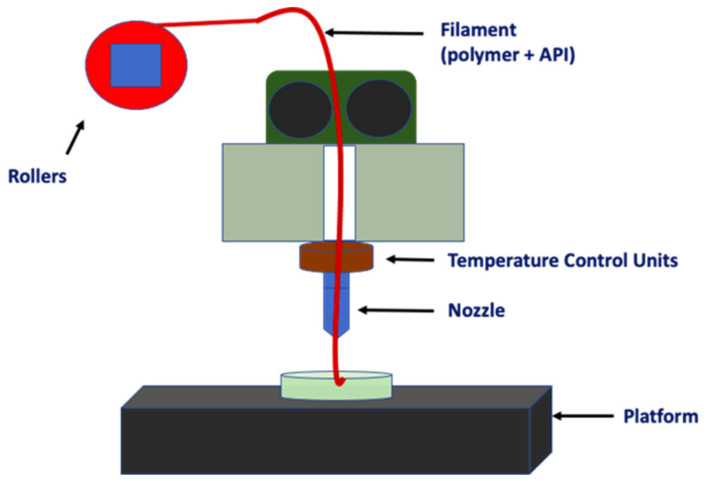
Fused Deposition Modelling (FDM) principles.

The FDM technique implies a fusion between material layers by depositing layers of thermoplastic material one-by-one. The material extrusion is the base of the FDM technique, in which the thermoplastic filament is heated until it melts. The melting process is carried out by a heated nozzle, which is positioned on the platform surface. This nozzle is part of the extruder head, and it is fed with the thermoplastic material through rotating rollers. This heating method is characterised by following a desired geometric pattern of the object, leaning on a computer-aided design (CAD) model [28] (Figure 2). Although FDM is widely used to produce solid models, it can be adapted to fabricate porous structures. In order to do this, a positive value could be applied to the raster fill gap, to impart a channel within a build layer. In this sense, the channels could be interconnected even in three dimensions when arranged in a regular manner [29].

The FDM process includes at least four stages: CAD modelling, pre-processing on FDM software, part building on an FDM machine, and post-processing of fabricated parts. As a first step, the process creates a 3D solid model in the CAD system and then converts it into an STL format, which can be processed by the FDM software. The parameters that might be included during the FDM process are the raster width, raster angle, air gap, build style, nozzle tip size, and temperature. Once the model file is sent to the printer machine, the printing process begins following the procedure previously described. After completion of the printing, the part could be removed from the printer. Finally, the support structures could be removed by breaking them from the main piece or immersing the model into different types of solutions in order to detach the support structures [30].

#### 2.1.2. FDM Materials

FDM techniques are most popular due to their material choice, which does not require the use of any toxic glues or solvents and has an accessible size with economics parts. In addition, thermoplastic polymers are the most common polymers used for printing, which allows an easy choice of scaffold components [31,32]. These materials include polyolefins such as polyethylene and polypropylene, polylactic acid (PLA), acrylonitrile–butadiene–styrene (ABS), polysulfone, polyetherimide, polycarbonate (PC), polyglycolic acid, polycaprolactone (PCL), and chitosan [33,34]. Among them, PCL, ABS, and PGA are the most widely used for skin, bone, and tendon repair [35,36,37], and the biochemical properties could be improved by the addition of different materials, such as β-tricalcium phosphate and hydroxyapatite [38]. The preference of ABS is due to its high strength, its resistance to corrosion, and low cost, which allows ABS to be used in conditions where other materials are not compatible. Additionally, it is possible to get ABS plastic in different colours, being a distinct characteristic. On the other hand, PC is also a widely used material, commonly applicable to the medicine and automotive and aerospace industries, among others. In addition, an advantage of this material is that it has better mechanical properties compared to ABS and other thermoplastic materials [39].

#### 2.1.3. FDM Parameters

During the FDM process, it is important to take care of all parameters that allow the control of shape, size, and internal structure of the part. Users could select the most important parameters such as build orientation, layer height, model build temperature, nozzle diameter, infill style, part interior density, raster width, raster angle, and air gap [40].

**Figure 3:**
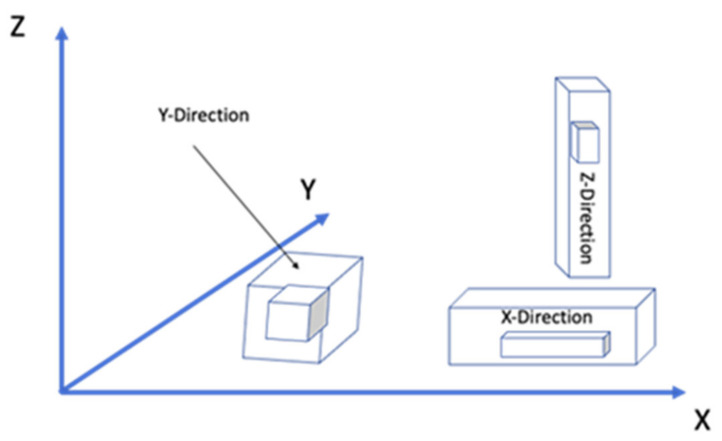
Build orientation.

**Figure 4:**
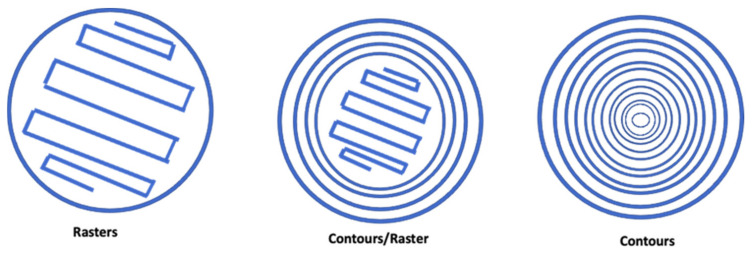
Types of infill styles.

**Figure 5:**
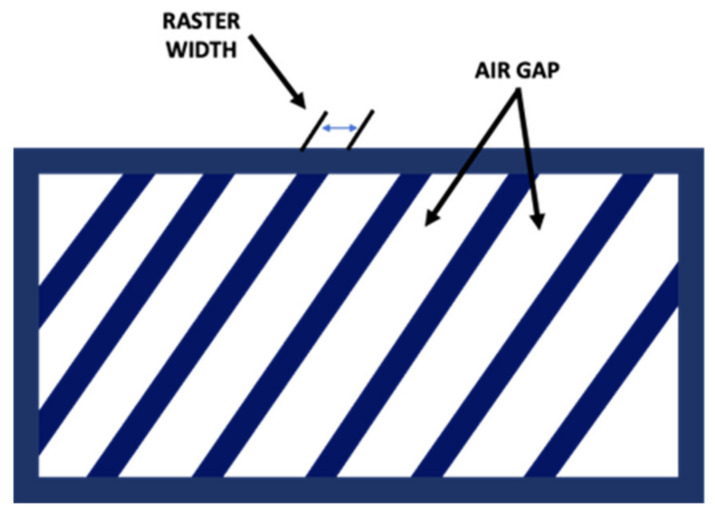
Raster width and air gap.

**Figure 6:**
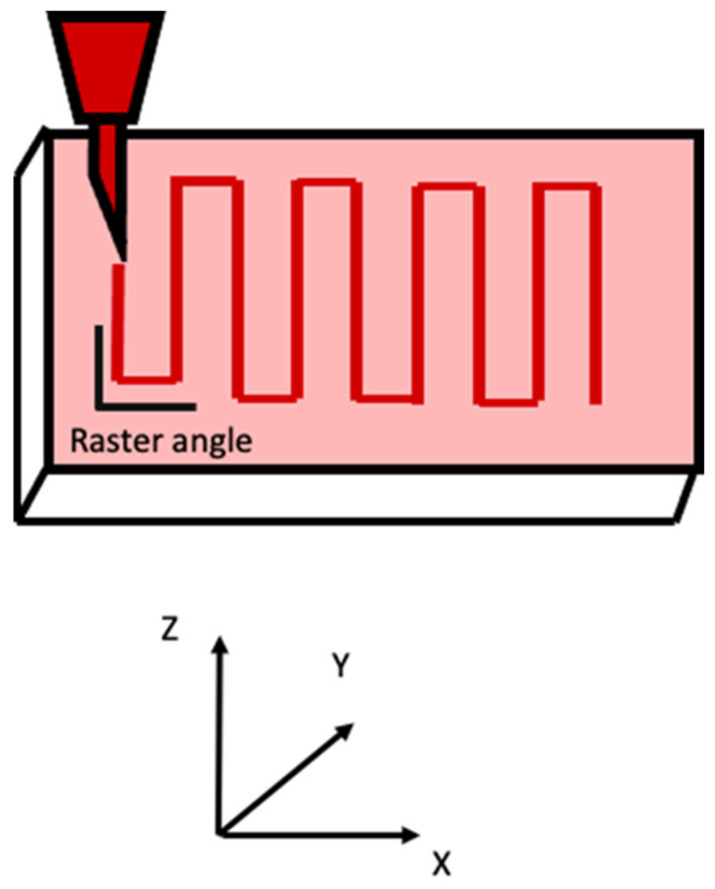
Raster angle.

- Build orientation: the way in which the component could be adjusted into the building platform using the three axes: X, Y, and Z of the FDM machine (Figure 3).
- Layer height: referred to as the layer thickness and the amount of material that is deposited along the z-direction. This parameter impacts directly into the building time and the quality of the surface. In addition, the layer height depends completely on the extruder tip diameter.
- Model build temperature: the temperature of the material in the heating nozzle. This temperature regulates the material viscosity extruded from the tip.
- Nozzle diameter: since it affects the drop pressure, it directly regulates the road width. In order to maintain a consistent flow of the extruding material, the correct nozzle diameter is required. In this sense, a different range of tips is often provided by FDM systems. The smallest nozzle diameters need more time to complete the extrusion process.
- Infill style: allows determination of the internal pattern of the structure, which could be raster, contour, or contour–raster. The most frequently used is the raster fill style, produced by the nozzle movement back and forth to fill the delimited area. On the other hand, to produce contour style, the tip movements are as a closed-loop. Finally, the combination of both previous approaches allows one to get the contour–raster style (Figure 4).
- Part interior density: associated with the air gap inside the raster and it gives information about the density of the material. Three types are available: solid, sparse, and sparse-double dense. In the first one, no air is inside the material. On the other hand, the sparse type allows a specified air gap between the tool paths. Finally, the sparse-double type is similar to the sparse type with the addition that it produces a hexagonal pattern.
- Raster width: the thickness of the material deposited from the tip to the platform. It depends on the tip size (Figure 5).
- Raster angle: the direction that the raster tool path is deposited on the *x*-axis, which could vary from 0° to 90° (Figure 6).
- Air gap: the space between two tool paths. Among the air gap types, the most common are: raster-to-raster air gap, used with the adjacent raster tool path with the solid infill style; part sparse, commonly used with the sparse infill style; and perimeter-to-raster air gap, used to describe the gap among the inner contour and the edge of the raster fill inside the contour (Figure 5).

#### 2.1.4. FDM Advantages and Disadvantages

Finally, it is important to highlight the advantages and disadvantages of the FDM technique. Among the first ones, an interesting advantage is that FDM is simple and safe, since it does not use toxic materials and is easy to use. In addition, once the printing has finished, the only additional step is the support removal, but besides that, it could be handled right afterward. Another important advantage is that the exact amount of material is used due to its extrusion process, avoiding material waste. As it was mentioned previously, users could produce solid or porous parts, since the software allows modifications of various of parameters, such as fill pattern, raster width and angle, and air gaps. Regarding tissue engineering, this method has the advantage of being very simple [25]. Moreover, parts could be printed using different materials and include the possibility of adding new types of materials as long as they meet the necessary requirements. In this sense, bioinks are commonly composed of hydrogels and some bioactive components with shear-thinning or fast-solidifying properties to produce the 3D structures with high accuracy [41]. In addition, there are important advantages regarding the bioprinting process. Among them is the possibility of printing high-viscosity bioinks and high concentrations of cells when processed at physiological temperatures [42].

On the other hand, there are some disadvantages, such as accuracy, since in some cases, the parts could present a grainy surface due to the layer-by-layer deposition process through the nozzle. In addition, it could take a long time to get the part done because of the slow printing speed, which is a consequence of having only one nozzle tip to make the layers. Finally, every new material should meet the diameter requirements of the nozzle tip to be used in the FDM printer [39]. Regarding the printing of biomaterials, the major limitation is the material selection, since the high temperature used during the process does not allow the cell printing, which requires a second step to seed cells on the constructs [38]. Another drawback is the resolution, since it is lower than other methods [43]. Last but not least, the materials could produce the clogging of the nozzle [44].

### 2.2. Stereolithography (SLA)

Steroelithography is a process based on photopolymerisation that uses a laser beam to print a particular model on a photosensitive resin [45]. The SLA technique is widely used in the industry since it is the oldest one. In addition, it could be used in different applications, from prototyping consumer products to printing tissues [46]. Indeed, some authors reported the fabrication of an optimal vascular network for tissue engineering skin using SLA technology with biocompatible, elastic, and surface-coatable materials [47].

#### 2.2.1. SLA Process

**Figure 7:**
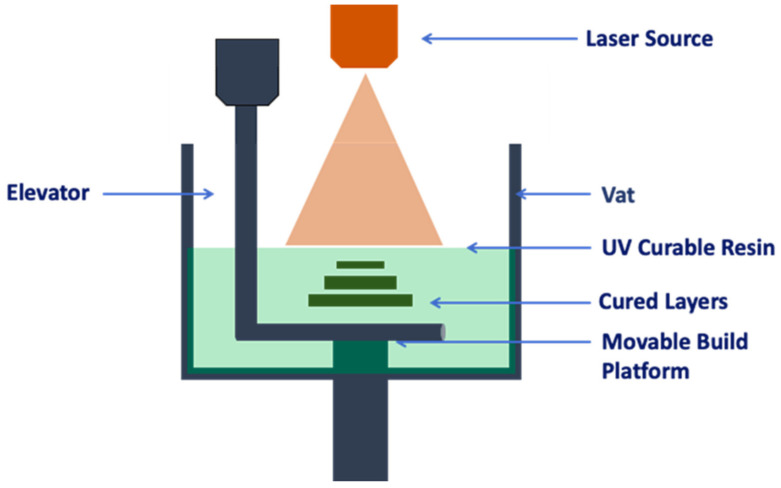
Stereolithography (SLA).

**Figure 8:**
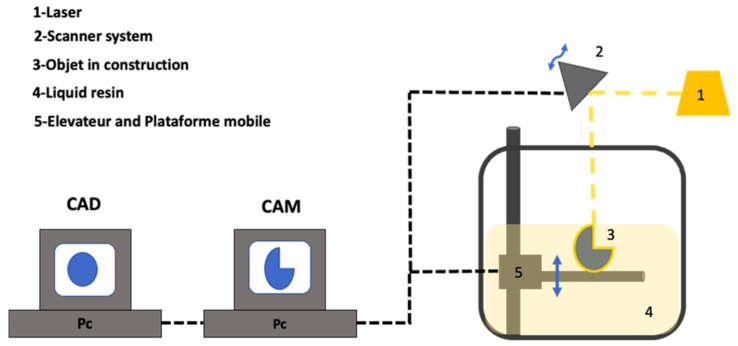
SLA process.

A UV laser (355 nm) was used in an SLA process in order to solidify a UV-curable resin using photopolymerisation (Figure 7). The pattern is designed by a computer-controlled laser beam or by a digital light projection on the resin surface. The printing process begins when the platform is immersed below the surface of a tank full of the liquid resin (pre-polymer solution). After that, the resin is solidified by the laser beam with the desired pattern. Once the layer is photopolymerised (solidified), the platform is lowered in order to deposit the following layer. The laser beam movement controls the pattern formation and, since it could move across a large space, it is able to produce large-size models [48] (Figure 8).

#### 2.2.2. SLA Materials

The material used in the SLA technique should be a photosensitive resin. Cationic photopolymerisation or free radical photopolymerisation are the mechanisms used by the photosensitive resin in SLA. The use of these resins is based on the basis that at 355 nm, both radical and cationic photopolymerisation could occur. The most common materials used, such as epoxy, thermoplastics elastomers, or acrylate resins, have interesting properties such as low viscosity and high photosensitivity. In this sense, these materials also have controllable mechanical properties and can stand changes of temperature and humidity. However, a major disadvantage is their high-volume shrinkage, which limits their use. In this vein, cationic photopolymerisation has no volume shrinkage. Among the cationic photoinitiators, the most common structures found are diazonium salts, diaryliodonium salts, triarylsulfonium salts, ferrocenium salts, and thiopyrylium salts. However, the resins used for cationic photopolymerisation are less and the initiator price is high; therefore, the hybrid photosensitive resins (radical and cationic) are the most commonly used [49,50]. The cationic photopolymerisation begins when the UV light absorption produces a homolytic and heterolytic cleavage of the salt. The products, cationic and cation-radical species, react and form strong protonic acids. These substances are responsible for the beginning of the cationic polymerisation since these species produce the direct protonation of the monomer. Acrylated polycaprolactone, gelatin methacryloyl, poly (propylene fumarate), and soybean-oil-epoxidised acrylate are the most common materials used in SLA printing [51].

#### 2.2.3. SLA Parameters

There are some parameters to take into account during the SLA printing process. These are part, support, and recoat parameters. Part parameters are the ones that could affect the accuracy of built parts. In this vein, the parameters included are [52]:

- Layer thickness: the layer depth.
- Hatch spacing: the distance between adjacent strands.
- Fill spacing: when the strand is at the part top or bottom surface.
- Overcure: the depth at which a strand penetrates the lower adjacent layer.
- Fill cure depth: the strand’s depth.

Other parameters are related to the shrinkage during the post-curing process. The post-cure shrinkage amount is due to the degree of the prototype cure in the green state, achieved during laser scanning. It is known that when the degree of curing of the prototype increases, the contraction is reduced. The cure degree also depends on [53]:

- Laser power: when it increases, the curing degree is higher. This occurs because when the laser has a high power, the resin is exposed to higher UV light intensity, which produces more crosslinking.
- Layer pitch: the curing degree is lower with a higher layer pitch, due to the fact that a lower layer pitch increases the overlaps between adjacent layers, decreasing the amount of uncured resin.
- Scan pitch: with a higher scan pitch, the curing degree is lower, since the uncured resin is higher.
- Scan speed: if the laser scan is faster, the curing degree decreases, since the exposure energy per unit area is less.
- Laser stability: when the laser power has any fluctuation, it leads to different laser exposures, which affect the curing degree.
- Absorption rate of the materials: the curing degree improves when the material absorption rate is higher.

#### 2.2.4. SLA Advantages and Disadvantages

SLA printing presents a wide range of advantages, such as a stable printing process and the highest resolution compared to other printing techniques. This is due to the resolution reached by SLA printers, which is 20 µm or less, when the other printers’ resolutions are between 50 and200 µm [54,55]. Another advantage is the possibility of printing large-size models. However, since the printing rate depends on the laser beam movement, the larger the size of the models, the slower the printing rate is [56]. In this vein, the printing process of the SLA technique is usually slow due to the low photopolymerisation rates during the printing process. In addition, the process is called “discontinuous” since there are different steps, such as the laser scanning, the movement of the platform, and the resin refill, which are separate. Between these steps, there is a time during which there is no printing. Another important disadvantage is that some biocompatible resins are unable to be used in this printing system and it is incapable of printing cells [57,58]. This is due to the UV irradiation, which could damage the DNA and promote the lysis of the cells [59].

### 2.3. Selective Laser Sintering (SLS)

The SLS technique implies the use of a high-powered laser to produce 3D parts from CAD geometry [60]. In SLS technology, the geometric complexity of the part is not important and parts could be produced by adding the material layer-by-layer [61]. In this vein, in order to produce functional plastic components, SLS is the most common technique [62]. This technique could be used to print acellular scaffolds. In this sense, the use of biocompatible and biodegradable polymers allows printing bone scaffolds with the SLS method [63].

#### 2.3.1. SLS Process

**Figure 9:**
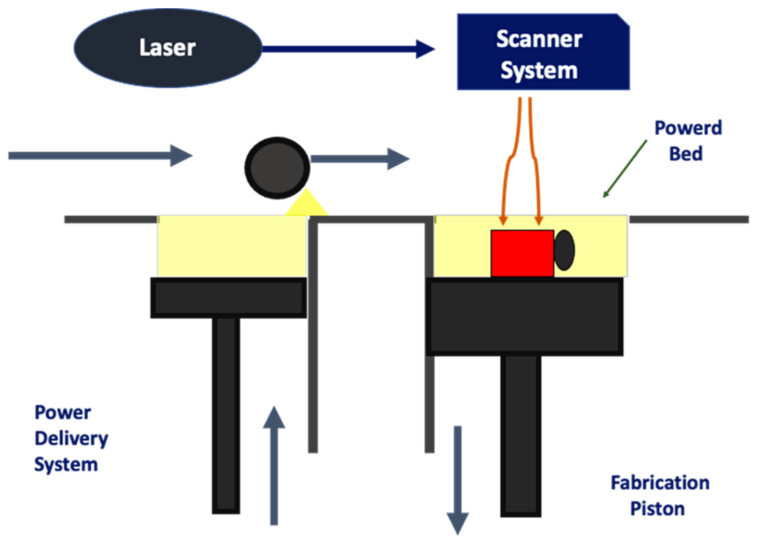
Selective Laser Sintering (SLS).

In this printing process, the scaffold is produced layer-by-layer. The use of a CO_2_ laser beam turns the powder into solid objects by fusing powdered, polymer-based materials such as nylon or polyamide [64]. The printing process begins when the laser crosses the powder in both axes: X and Y, building a profile with two dimensions. The interaction between the laser and the powder causes an increase of the temperature up to the melting point, leading to the fusion and the resulting mass. This process is called sintering [1]. The printing continues when the printer platform lowers, and a new layer of powder is distributed. (Figure 9).

#### 2.3.2. SLS Materials

In SLS, the most important materials used are polymers. In the printing process, three main types of polymers are used, namely, thermoplastics, thermosetting plastics, and elastomers. Thermoplastics are the most commonly used in the SLS technique. These thermoplastic polymers can be classified in the following: amorphous and crystalline. Both materials have particular properties that should be taken into account while setting the parameters. In this sense, the crystalline material’s chain molecules are arranged in an orderly structure, whereas the amorphous material’s chain molecules are disposed in a random manner. These differences in the chain molecule arrangement lead to different thermal properties [65]. Among the widely used materials in the SLS printing process, the most common are PC, ABS, polyamide, poly(L,D) lactic acid-bioactive glass, polylactide–calcium carbonate, poly(3-hydroxybutyrate-co-3-hydroxyvalerate), polycaprolactone–hydroxyapatite, poly(D,L-lactide)-β-tricalcium phosphate, polyamide–hydroxyapatite, and titanium [66,67,68].

#### 2.3.3. SLS Parameters

**Figure 10:**
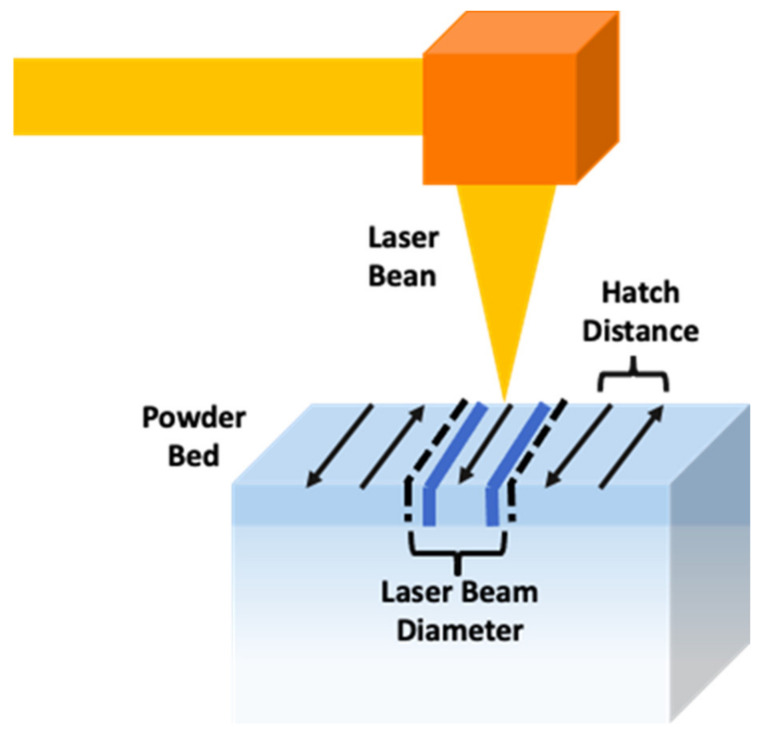
SLS parameters.

It is important to take into account the software parameters before the printing process. These parameters could vary according to the properties of the material powder used. Among them, the most important are [69] (Figure 10):

- Part bed temperature: the temperature that controls the powder into the part cylinder. The powder is heated in the part cylinder before the movement of the laser scanners, and the part bed temperature is important to reduce laser powder and distortion.
- Fill laser power: the power of the laser beam at the part bed surface. This parameter should be set in order to ensure that the powder would heat up to the melting temperature in the part bed surface.
- Scan size: sets the speed of the laser beam.
- Scan spacing: the space between two neighbouring parallel scan vectors. It is associated with the size of the laser beam and the energy density.
- Slice thickness: it is the powder thickness of each layer in the part cylinder. It depends on the part piston depth when it lowers.

#### 2.3.4. SLS Advantages and Disadvantages

The major advantage of the SLS method is its high fracture toughness and mechanical strength, providing high quality for implants [70]. Another important advantage of this technique is the possibility to create components without the supporting structures. In this sense, in each build more parts could be produced, reducing the required amount of post-processing. However, the part strength could be inconsistent, leading to the possibility of different strength for multiple copies of the same part [62]. The wide range of available biomaterials is an advantage of this technique for tissue engineering applications. In this sense, bone replacements or structural-supporting materials could be fabricated using ceramics and metals [71]. Compared to conventional techniques, tissue regeneration could be improved because of the controlled pore size of the scaffold [72,73]. However, because of the high temperature reached during the radiation of the CO_2_ laser, cells could not be printed and thermally stable polymers are required [74].

### 2.4. Low-Temperature Deposition Manufacturing (LDM)

In order to fabricate different scaffolds, LDM printers use a more robust technology, when compared to the previously described printers, such as FDM and SLS [75]. The bioactivity of the different materials is preserved thanks to its non-heating characteristic [76]. Natural biopolymers could be printed maintaining their bioactivities. The 3D structures are fabricated with FDM technology, by consecutive addition of extrudate layers following a computer design model [77]. In fact, it was described that the fabrication of a bilayer scaffold for skin tissue engineering applications was made with the upper layer of poly(e-caprolactone-co-lactide)/Poloxamer (PLCL/Poloxamer) nanofibre membrane, and the lower layer was a hydrogel composed of 10% dextran and 20% gelatin [78].

#### 2.4.1. LDM Process

**Figure 11:**
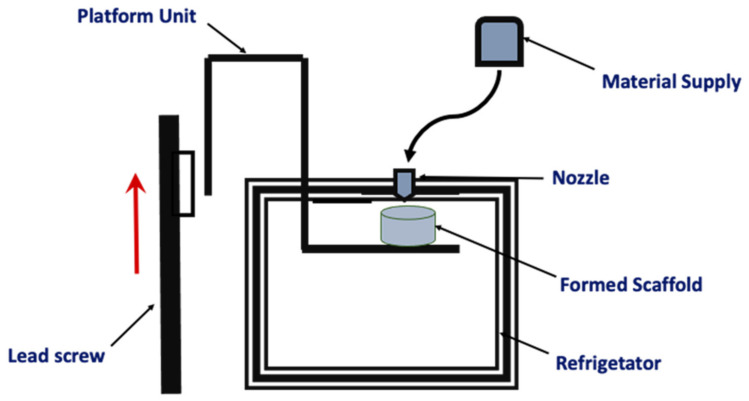
Low-temperature deposition manufacturing (LDM).

The LDM printer fabricates the scaffold using a chamber where the temperature does not exceed 0 °C. The platform used to produce the scaffold is inside this chamber. Using a layer-by-layer method, the scaffold is produced, and it is freeze-dried in order to remove the frozen solvent. In this sense, the LDM technique implies the manufacturing process with the addition of the phase separation process [79] (Figure 11).

#### 2.4.2. LDM Materials

The LDM printing process allows the use of natural biopolymers, such as collagen type I, sodium alginate, gelatin, and chitosan. In this vein, these materials can keep their bioactivities thanks to the non-heating property [80]. In addition, in order to improve the mechanical and biological properties of the scaffold, inorganic particles could be added. Among these particles, the most frequent are nano-hydroxyapatite, tricalcium phosphate, and magnesium particles [81]. In this vein, the LDM technique allows the production of different scaffolds.

#### 2.4.3. LDM Parameters

**Figure 12:**
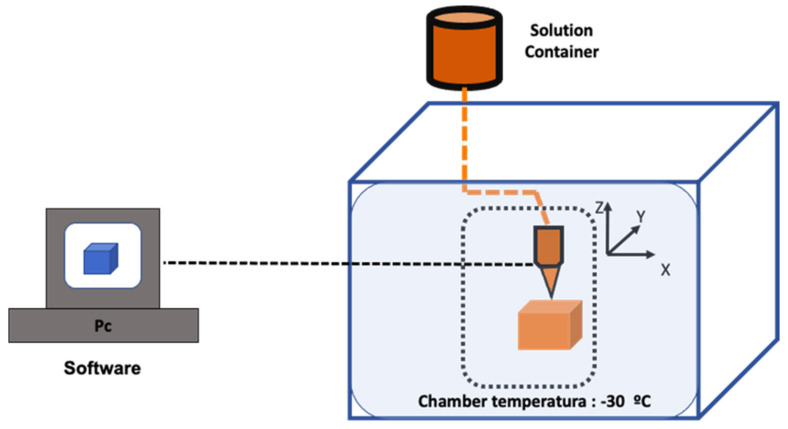
LDM parameters.

In order to produce the correct printed part, it is important to adjust the LDM parameters. Among them, the most common are [76]:

- Software: it is necessary to design the electrical model, determining the shape and architecture of the part.
- Material properties: related to the built part morphology and structure. In this vein, when scaffolds are printed, it is important to consider that their structure depends on the proportion of materials.
- LDM device parameters, which include the following parameters:
  - The chamber temperature: it should be around −30 °C in order to ensure that the extruded material is frozen.
  - The nozzle temperature: needs to be higher than the previous one to ensure that the extruded lines could integrate with the previous layer.
  - Nozzle diameter, nozzle scanning speed, and extrusion rate: define the extruded slurry lines’ morphology and diameter. A lower extrusion rate and higher nozzle scanning speed could decrease the line’s diameter, leading to broken lines.
  - Material solution viscosity: determines the morphology and the final structure of the built part. It is also related to broken lines, since a material with high viscosity is difficult to squeeze out of the nozzle. However, it is possible to improve this problem by increasing the nozzle temperature (Figure 12).

#### 2.4.4. LDM Advantages and Disadvantages

An important advantage of the LDM technique is the high versatility due to the fact that viscous liquids could be prepared even at room temperature. However, it is difficult to reach appropriate flow parameters while selecting the concentration of the solvent and polymer without affecting the solvent’s rapid evaporation in the solution [82]. Optimisation of printing parameters is required to achieve the desired extrusion of the dissolved polymer. In addition, the selection of the proper solvents applicable to the polymers is critical to achieve correct liquid viscosity without altering the rheological requirements [83]. Another characteristic of this printing technique is the importance in maintaining the chamber temperature around −30 °C, while the temperature of the nozzle needs to be higher [84].

### 2.5. Laser-Based Bioprinting

The laser-based bioprinting components are the laser source (which could be pulsed or continuous), a laser transparent print ribbon (which may contain a laser-energy-absorbing layer) coated with the layer of cell-laden bioink, and the collector slide that is on a motorised stage. The cell-laden material is patterned in a three-dimensional spatial arrangement by the energy from the laser using the computer design (CAD/CAM) [85]. During the process, the energy-absorbing layer is stimulated by a focused laser pulse that came from the laser source. The energy absorbed vaporises the donor layer, creating a high-pressure bubble that pushes the bioink as droplets into the receiving substrate. This method has a high resolution and reproducibility, making it available to print stem cell graft and skin tissue, among others [86]. In order to produce high-quality products, it is important to consider the laser’s wavelength, intensity, and pulse time. In addition, surface tension, viscosity, and tension are also key for the bioinks. Finally, the air gap between the “ribbon” structure and the substrate is also important parameters to be considered [87,88].

Among the different laser-based bioprinting techniques, the most common ones include laser-induced forward transfer (LFT), absorbing film-assisted laser-induced forward transfer (AFA-LIFT), biological laser processing (BioLP), matrix-assisted pulsed laser evaporation direct writing (MAPLE-DW), and laser-guided direct writing (LG DW) [89].

The laser-based bioprinting was used in the fabrication of multi-layered tissue constructs, such as skin tissue using fibroblast and keratinocytes in collagen that could mimic the tissue functions [90,91]. One of the most important advantages of this printing technique is the non-contact process which eliminates the nozzle clogging. In addition, this technique presents a high resolution (50 µ), since it presents the capability of printing single cells per droplet, the possibility of using high-cell densities (10^8^ cells/mL), and low-viscosity cell suspensions (1–300 mPa s) [92,93]. However, some of the disadvantages include the risk of photonic cell damage due to the laser exposure; this is the most important. In addition, those using metals as a laser-energy-absorbing layer bring up the problem of cytotoxicity induced by metallic nanoparticles. Moreover, the scalability is limited due to the high cost of the laser system and the complexity of the control of the laser pulses [85].

### 2.6. Droplet-Based Bioprinting

In the droplet-based bioprinting technique, cell-laden bioinks are ejected out of the nozzle into a pre-defined location on the substrate, in the form of droplets [94]. They can be classified into inkjet bioprinting (continuous and drop-on-demand thermal, piezoelectric, and electrostatic), electro-hydrodynamic jetting (EHD jetting), acoustic bioprinting, and microvalve-based bioprinting [89].

The inkjet printing technology was adapted in order to assess the inkjet bioprinting, in which the printing ink cartridges are replaced with cell-laden bioink cartridges. This technique can be classified into two groups: continuous inkjet (CI) and drop-on-demand inkjet (DOD) printing. Among them, DOD printing is preferred for bioprinting since, due to the nature of the CI method, the droplet could not be precisely controlled [95]. In this sense, in the DOD method, a trigger ejects droplets on demand, leading to a precise control and positioning of droplets. The DOD bioprinting could be classified as thermal, piezo-electric, or electrostatic systems. All the systems allow printing cell-laden bioinks with a high post-printing cell viability [20,96,97,98,99,100]. In general, inkjet bioprinting was used to print tissue constructs of skin, among other tissues, such as bone, cartilage, cardiac, and nervous [101,102,103,104,105,106]. An important advantage of the inkjet bioprinting technique is the high resolution (50 μm), high printing speed (10,000 droplets per second), and the possibility of introducing cell concentration gradients [20]. On the other hand, some disadvantages are that only low-viscosity bioink (3–12 mPa s) could be printed, due to the nozzle clogging that limits the cell concentration in the bioink up to 106 cells/mL [93].

Electro-hydrodynamic jetting-based bioprinting allows the printing of living cells, such as Jurkat cells, mouse neuronal cells, human embryonic kidney cells, and mouse fibroblasts, beyond the high electric fields and forces associated with this process [107,108,109]. An important advantage of this technique is the high resolution (100 nm), since nanoscale resolution could be achieved and bioinks with high viscosity (1–1000 mPa s) can be printed [107,110]. However, an important disadvantage it that the exposure to the high voltage and high electric fields could be detrimental to the cell viability on the long-term post printing [111].

In the acoustic bioprinting method, cell-laden bioink droplets can be ejected on demand. This technique allows the bioprinting of different types of cells, including mouse embryonic stem cells, fibroblasts, hepatocytes, human Raji cells, and HL-1 cardiomyocytes [112]. An important advantage of this method is that the bioink is in an open pool instead of being in a nozzle, avoiding some stressors, such as heat, high pressure, and voltage [95]. In addition, this technique has a high resolution (37 μm) and high printing speed (10,000 droplets per second). However, this method does not allow bioinks with high viscosity and high cell concentration [94].

In the microvalve-based bioprinting, to control the droplet ejection of cell-laden bioink, electromechanical or solenoid valves are used [113]. This method was used to print different types of cells, such as fibroblasts and keratinocytes, primary bladder smooth muscle cells, and human alveolar epithelial type II, with an interesting post-printing cell viability [91,114]. In addition, multi-layered skin tissues and lung tissue analogue constructs were printed using this technique [91]. An important advantage is the possibility of synchronised ejection from different print heads, allowing the co-culture printing and multi-culture tissue constructs [113]. Another advantage is that the cells are less likely to be damaged since the pneumatic pressure used is lesser than the one used in inkjet bioprinting [95]. Among the drawbacks, the printing speed is moderate (1000 droplets per second), it has a low resolution compared to other methods, the viscosity is limited (1–200 mPa s) due to the nozzle clogging, and the cell concentration is fewer than 106 cells/mL [91].

In general, among the advantages of the droplet-based bioprinting, the most important one is its compatibility with a variety of biological materials. In addition, this technique provides a high resolution (20–100 μm) and speed (1–10,000 droplets/s) while being an interesting low-cost possibility [115]. However, an important disadvantage is the requirement of a liquid or less viscous form of the biological material.

### 2.7. Extrusion-Based Bioprinting

In the extrusion-based bioprinting method, pneumatic pressure or mechanical force are used to extrude the bioink out of the nozzle in an uninterrupted line [89]. This technique originates from fused deposition modelling (FDM) printing.

Extrusion-based bioprinting was used to bioprint cells, tissues, organ modules, and organ-on-a-chip devices, for tissue engineering, cancer research, drug testing, and transplantation [116]. Among the types of tissues printed, some are skin [117], bone [118], cartilage [119], skeletal muscle [120], cardiac tissue [121] and nervous tissue [122]. In addition, cell-laden constructs had gelatin methacryloyl, an alginate core, and sheath microfibres [123].

A major advantage of this method is the scalability, since it has a continuous bioink flow and large deposition rate. In addition, it allows high viscosity bioinks (600 kPa s) and high cell concentrations (10^8^ cells/mL) [42]. Depending on the bioink viscosity, cell concentration, and nozzle size, post-printing cell viability could be around 40% and 95% [93]. The requirement of bioinks with shear-thinning properties is another drawback for this technique. On the other hand, this method presents a lower resolution (100 μm) than the others [43,124], and another disadvantage is the nozzle clogging [44].

### 2.8. Stereolithography-Based Bioprinting

Stereolithography-based bioprinting uses a light irradiation, commonly UV, to polymerise a layer of photopolymer resin. A computer code controls the light movement in order to form the 3D structure as the build stage is translated, vertically building the construct layer-by-layer [25]. The stereolithography method could be divided into two modalities; in one, a computer controls the light source and it moves towards the structure required in each layer of the 3D object. The other modality uses an array of several thousand micro-mirrors called a digital micromirror device (DMD). In this case, the micromirrors can be controlled in order to reflect the light in a spatial pattern, allowing the polymerisation of the whole layer at once [125]. This is an important advantage since it reduces the printing time.

Stereolithography bioprinting was used to bioprint murine embryonic fibroblasts and murine mesenchymal progenitor cells, human dermal fibroblasts, and embryonic dorsal root ganglia [59,125,126]. In addition, using this method of three-dimensional biodegradable poly(ethylene glycol)/poly(D,L-lactide), hydrogel structures were prepared [127].

This approach has important advantages due to its precise control on the deposition of biologicals and high resolution (200 nm-6 μm) in reduced printing time. In addition, it permits the use of a high cell concentration (>106 cells/mL) with no nozzle clogging problem [89]. However, among the disadvantages, only the use of photocurable bioinks is the most important. In addition, the UV light can alter cells’ viability since the irradiation provokes damage of the DNA and promotes cell lysis, and only low viscosity (5 Pa s) of bioinks could be used [89].

### 2.9. Printer Softwares

In the last thirty years, in conjunction with the advance of 3D printing technology, computer-aided design software packages were used for modelling structures previously printed. UG, CATIA, or ProE, among other customised software, are used for this first step. Then, an ST-format file, which contains all the information models, is exported to the 3D printing system to control the moving track of the printing device and construct the structure layer-by-layer.

According to Pakhomova et al., [128] the software is classified based on control tools, general computer-aided design (CAD), tools used to convert medical data to CAD formats, and only a few specialised research project tools. The process of bioprinting shows three distinct phases. In the first step, considered as a pre-processing phase, all the planning details are calculated. This pre-process includes imaging (CT, MRI, etc.) used for the analysis of the anatomical structure of the tissue. Then, a proceeding by CAD is carried out to translate the imaging data into a blueprint for bioprinting. The imaging data are transformed into cross-sectional layers of appropriate scale, such that the bioprinting device will be able to add them in a layer-by-layer fashion. This step is carried out by specialised software programs such as AutoCAD, SOLIDWORKS, and CATIA, among others.

Subsequently, the processing phase is carried out and involves all steps related to construction and manufacturing of the bioprinted tissue. Complexity at this stage is related to the specific printing method and the combination of materials (bioink, scaffold, and other additives). Finally, the post-processing phase includes all steps that occur before bioprinted tissue is completely mature and ready to use [128,129].

## 3. Bioinks for 3D Printing Technology

In the past few years, the development and characterisation of new bioinks gained increasing attention, mostly because of the lack of materials suitable for bioprinting. This issue was considered as one of the major drawbacks that substantially limited the progress in the field. Therefore, the number of additive manufacturing techniques able to be used for 3D bioprinting increased over time, with the aim to include droplet deposition such as inkjet, extrusion, and microvalve-based techniques, and lithography and laser-forward, transfer-based techniques for tissue engineering purposes. All of them possess distinct physical and rheological requisites for a suitable ink [9].

The bioprinting process permits the fabrication of 3D tissue constructs with the previously programmed geometries and structures containing biomaterials and/or cells (together known as bioink), by synchronising the bioink crosslinking/deposition with a motorised stage movement. Despite the 3D bioprinting modality used, the bioinks are an essential component during the construct fabrication and they could be stabilised or crosslinked during or immediately after bioprinting to create the final shapes of the intended tissue constructs. The selection of the bioink depends on the specific application, for example, the target tissue, the cell type, and also the bioprinter that will be used [130].

Bioinks should fulfil several requirements to guarantee the success in the fabrication of tissue constructs. They must be highly biocompatible to accommodate live cells and mechanically stable after printing. Moreover, the bioink printability is mandatory. It depends on different parameters such as the surface tension of the bioink, the viscosity of the solution, and the capability to crosslink on its own and on surface properties of the printer nozzle. Furthermore, the printing reliability and encapsulation of live cells deeply depends on the viscosity and the hydrophilicity of the bioink. Some other important desirable aspects for a bioink to highlight include high resolution during printing, ready availability, low cost, their ability of biomimicking the tissue’s internal structures, and immunological compatibility [131]. In this sense, naturally derived biomaterials afford a good environment for cell growth by mimicking the native ECM of tissues, self-assembling, and showing biodegradation and biocompatibility properties. Nevertheless, they do not have the mechanical properties needed to conserve the integrity in the in vivo microenvironment and can be unstable and unpredictable. Moreover, poor mechanical properties may cause difficulties in printing, low, rigid tissue structures, and lesser support for the cells in the tissue [132]. Because of this, extensive research is being held, in order to optimise and improve the naturally derived biomaterials properties for their use in 3D printing. In this review, we focus on the most representative and common polymers used as bioinks for 3D bioprinting. Table 1 summarises detailed characteristics and advantages of various printing technologies used with collagen, chitosan, cellulose, hyaluronic acid, and alginic acid-based bioinks.

### 3.1. Collagen

Collagen is a protein which is the main component of the extracellular matrix of animals, representing approximately 30% of the protein content in vertebrates. It carries out a structural and functionality role. Tissue integrity within the body is assured given its strength and/or flexibility and stability [147]. Foreseeing the therapeutic benefits of collagen biomaterials and their association within composites or hybrids, a wide diversity of biomaterials have been prepared over nearly two decades [148,149,150,151].

Proteins are particularly interesting in the formulation of inks for 3D printing technology. They are essential structural components of living systems, providing support in and around cells, and they are important for tissue functions [152]. The skin, for instance, has a challenging, complex structure to bioprint, consisting of two major compartments: epidermis, dermis, and a third region known as the subcutaneous tissue [153,154]. Because of this, tissue-engineered skin remains elusive despite extensive research, due to the skin’s multi-stratified anisotropic structure. It is difficult to replicate applying traditional tissue engineering techniques [4]. Taking into account the skin tissue complexity, Park et al. [155] obtained 3D cell-laden collagen microstructures by 2D cell patterning. This technique provides a simple and powerful manner to mimic the functions and structures of complex tissues and organs. It also contributes to reducing the gap between human body and in vitro tissue models. They adapted this technique to fabricate human skin models with papillary structures at the dermo–epidermal junction. Throughout their study, they fabricated self-organised, 3D-protruded collagen microstructures by seeding fibroblasts within a hydrogel in patterns using inkjet cell printing. By studying the printing parameters, the collagen bed condition, and the cell number in a droplet, fibroblasts could be aligned in patterns with controlled cell numbers. Within the collagen matrices, fibroblasts rearranged and reorganised the surrounding extracellular matrix microenvironment. Moreover, vertically elevated collagen microstructures were formed relevantly to the size and the shape of the printed cell patterns.

Regardless of the bioprinting technology applied, the functionality of the bioprinted skin substitute is highly dependent on the bioink composition and cell type, in terms of rheology, mechanical integrity, biocompatibility, biodegradation, and antimicrobial activity [134]. In reference to collagen, many strategies were carried out to improve the integrity of collagen for printing purposes. We can mention the following: (i) changing collagen properties thanks to additives, inducing partial crosslinking, or chemical modification; (ii) printing collagen into a support such as a thermoplastic scaffold or slurry baths; or (iii) using collagen as the binder/crosslinker [156]. In this sense, Shi et al. [157] prepared a novel bioink constituted by gelatin methacrylamide (GelMA) and collagen doped with tyrosinase for the 3D extrusion-based bioprinting of living skin tissues. Tyrosinase has a dual function since it is an essential bioactive compound in the skin regeneration process, but also an enzyme that facilitates the crosslinking of collagen and GelMA. The crosslinking strategy was adopted to enhance the bioink mechanical strength and printability. In vitro cell culture results have shown that tyrosinase favours human melanocytes proliferation and inhibits the growth and migration of human dermal fibroblasts. In vivo tests showed that the wound healing rates may be accelerated when treated with tyrosinase-doped bioinks.

Furthermore, Bell et al. [158] presented a method that allows multiphoton crosslinking of collagen type I with flavin mononucleotide photosensitiser. This method permits the full 3D printing of crosslinked structures using unmodified collagen type I and uses only biocompatible materials. Complex 3D structures were successfully fabricated, and they obtained a resolution of 1 μm for both standing lines and the high aspect ratio gap between structures. Their work details a 3D printing technique with one of the most widely used tissue scaffold materials: collagen. It is worth noting that high resolution and 3D control of the fabrication of collagen scaffolds facilitates recreation with a higher fidelity of the native extracellular environment for tissue engineering.

Additionally, Wei Long et al., [159] reported a single-step bioprinting process that may be useful for the fabrication of complex 3D tissue models for tissue engineering applications. It consists of a bioprinting-macromolecular crowding process (BMCP) and an additional printing cartridge consisting of 1 million fibroblasts/mL in PVP-based bioink that is used to print discrete cell droplets onto each printed collagen layer. Their results indicated that the number of living cells increased over a period of 10 days, indicating that the BMCP is biocompatible and does not exert detrimental effects on the printed cells. Moreover, ImageJ analysis of the stained living cells (cell perimeter and cell area) showed that the elongated fibroblasts are gradually spreading within the collagen matrix. These findings could be attractive for the structural design of collagen-based hydrogels for tissue engineering.

### 3.2. Chitosan

Chitosan is a biopolymer obtained from the deacetylation of chitin. It is a polysaccharide constituted by randomly distributed monomeric units of b-(1–4)-D-glucosamine and *N*-acetyl-D-glucosamine. This biomaterial is extensively used for tissue engineering purposes and lately, 3D printing of chitosan-based materials have been widely explored because of their excellent biodegradability as well as biocompatibility [141,160,161].

The skin is the largest organ of the body and the first line of defence against external factors including pathogens or mutagenic substances. Skin damage can be caused by any chemical, thermal, or electrical stimuli, and sometimes cutaneous complications or adverse reactions may lead to chronic and hard to heal injuries. In this matter, tissue engineering can provide a promising solution since it attempts to mimic the natural system morphology and, therefore, promotes an effective healing process.

According to a study by Smandri et al., [162] natural-based bioinks for three-dimensional bioprinting have an excellent ability to mimic the three-dimensional microenvironment structure of native skin tissue and to encourage cell adhesion, migration, proliferation, and mobility. Moreover, in vivo studies showed full wound closure four weeks post-surgery, with well-organised dermal and epidermal layers.

Regarding the chitosan biopolymer, it has been previously reported that chitosan-based functional constructs are appropriate for tissue engineering because chitosan is non-toxic, biocompatible, and biodegradable, and it can be modified to obtain multifunctional constructs that are similar to the natural matrix [163]. It is worth mentioning that for 3D printing purposes, chitosan hydrogels are not that suitable as ink of 3D printers to construct complex patterns, because the formation of chitosan hydrogel involves the neutralisation of chitosan acidic solutions. Nevertheless, by controlling the rheological property of chitosan solutions and solvent evaporation, the 3D printing of complex structures obtained of chitosan ink were reported [164].

In 3D printing technology, two aspects in the 3D ink development must be considered. Firstly, the hydrogel precursor features to accomplish proper injectability and shape fidelity to the digital design, and secondly, suitable mechanical properties of the hydrogel after crosslinking, to allow scaffold integrity and cell proliferation. In this sense, Heidenreich et al. [165] studied the rheological properties and printability of hydrogel precursors containing different proportions of chitosan (chi) and collagen (col), seeking proper inks for extrusion 3D bioprinting. Three inks with different polymer ratios (col:chi 0.18:1.50, col:chi 0.36:1.00, and col:chi 0.54:0.50), presented acceptable printability values under printing flows between 0.19 μL/s and 0.42 μL/s. The best formulation, col:chi 0.36:1.00, was chosen to print mono-layered scaffolds. They demonstrated stability after 44 h in a buffer of PBS with collagenase at a physiological level, and had no cytotoxic effect towards the NIH-3T3 fibroblasts.

An innovative extrusion-based 3D printing technique worth mentioning has been used by Intini et al. [26] for the preparation of novel 3D chitosan scaffolds presenting controlled and reproducible macro and microstructures to be applied in the regenerative skin tissue field. Their manufacturing approach combines the freeze-gelation method together with an advantageous modification of chitosan solution with raffinose. They evaluated the 3D chitosan scaffolds in terms of cytocompatibility, biocompatibility, and toxicity towards the human fibroblasts and keratinocytes. In vitro results showed that 3D cell cultures achieved after 20 and 35 days of incubation had significant qualitative and quantitative cell growth. Additionally, the tests of 3D printed scaffolds in wound healing performed on streptozotocin-induced diabetic rats demonstrated that 3D printed scaffolds improved the quality of the restored tissue in comparison to commercial patches and spontaneous healing.

Another approach to note is adding chitosan as particles in the bioink. For instance, Andriotis et al., prepared biodegradable 3D-printable inks based on pectin biopolymer as a system for direct and indirect wound-dressing applications, suitable for 3D printing manufacturing. The 3D-printable inks obtained formed free-standing transparent films upon drying, revealing fast disintegration upon contact with aqueous media. To enhance the antimicrobial and wound-healing activities of the inks, particles were added, comprised of chitosan and cyclodextrin inclusion complexes with a propolis extract. The in vitro studies exhibited that 3D-bioprinted patches enhanced the in vitro wound-healing process, while the incorporation of chitosan and cyclodextrin/propolis extract inclusion complexes further enhanced wound healing, and also the antimicrobial activity of the patches [166].

### 3.3. Celullose

Cellulose is an abundant bio-based homopolymer in nature. It plays a crucial role in preserving the structure of plant cell walls, is present in tunicates, and supports flocculation processes in bacteria, such as Acetobacter xylinum [167]. Cellulose is a water-insoluble polysaccharide composed of d-glucopyranose moieties joined by b-1,4 linkages by oxygen atoms [168,169]. Depending on how these chains of β-(1,4′)-D-glucopyranose are assembled, cellulose can have different structural allomorphs, i.e., cellulose I, II, and III [170]. Cellulose I is the natural form of cellulose composed of parallel glucose-based chains, giving two crystal structures: cellulose Iα that is present in high quantities in bacteria and alga, and cellulose Iβ that is predominant in higher plants. Cellulose II and III are synthetic-derived celluloses, the first one with an antiparallel arrangement and the second one characterised by hydrogen bonds between separate sheets.

Due to its diverse and tunable mechanical, structural, chemical, and physical properties, cellulose is a perfect alternative for a wide range of applications, especially for biomaterial fabrication for tissue engineering [171,172,173]. Besides, its high biocompatibility, changeable biomechanics, biodegradability, high availability in nature, and moisture conservation make cellulose-based bioink an effective and low-cost material for skin regeneration, drug delivery, and wound healing [140,142,162,174]. In this regard, in recent years, several researches that exploit cellulose to develop bioinks with good printability properties and bioactive characteristics have been reported [175,176]. For example, cellulose nanofibrils (CNFs) have been crosslinked with different metallic cations (Fe^3+^, Al^3+^, Ca^2+^, and Mg^2+^) to develop hydrogel-based inks for 3D printing applications [177]. For this, cellulose pulp was mechanically disintegrated and oxidised with 2,2,6,6-tetramethylpiperidine-1-oxyl (TEMPO) to obtain the CNFs. Then, the deprotonated, TEMPO-oxidized CNFs were crosslinked with the divalent and trivalent metal cations, and the corresponding hydrogel was formed. They found that by varying the nature of the cations they could modify the properties of the hydrogel-based inks. In fact, hydrogels containing divalent cations Ca^2+^ and Mg^2+^ had good 3D printing performance, while the hydrogels incorporating trivalent cations Fe^3+^ and Al^3+^ were unprintable. Gatenholm has also used cellulose nanofibrils to yield a bioink for 3D bioprinting of tissue and organs with a special design [178]. He introduced a novel bioink, CELLINKTM, composed of crosslinked nanofibrillated cellulose with desired morphological and rheological characteristics. In this invention, a purification step is crucial for adjusting osmolarity of the material and sterilisation to produce a cytocompatible biomaterial that can incorporate living cells, such as fibroblasts, chondrocytes, and stem cells. The biocompatibility and biomimicry properties of these new bioinks based on nanocellulose fibrils make them promising candidates for applications in cell cultures, tissue engineering, and regenerative medicine.

The advantage of designing a bioink composed of different hydrogels lies in the possibility of printing uniform 3D structures with high resolution and shape integrity. In this sense, Rastin et al. developed a cell-laden bactericidal bioink based on a hybrid methylcellulose/alginate hydrogel (MC/Alg) for skin regeneration [143]. The particularity in the design of this bioink was the use of gallium (Ga^3+^) after printing 3D structures by extrusion of the MC/Alg hydrogel. Immersion of the three-dimensional MC/Alg multilayered scaffolds in the Ga^3+^ solution led to stabilisation of cellulose-based bioink by crosslinking with alginate chains. Furthermore, due to the broad antibacterial activity of Ga3+, the gallium-crosslinked bioink demonstrated potent bactericidal action against both Gram positive and Gram negative bacteria. In addition, the bioink exhibited high biocompatibility, supporting fibroblast cellular functions. Taken together, the excellent printability, good rheological properties, effective bactericidal activity, and high biocompatibility make this MC-Alg-Ga bioink a potential candidate for skin tissue engineering. Zidarič et al. also combined cellulose-based materials with alginate to design a novel hybrid bioink for 3D bioprinting of a dermis layer [179]. To prepare the bioink, they mixed the viscoelastic CNFs with the fast-crosslinking Alg and carboxymethyl cellulose (CMC), and incorporated human-derived skin fibroblasts (hSF) before the extrusion process. In this case, to support cell proliferation after the 3D bioprinting, the designed bioink formulation must yield a quasi-scaffold structure, thus a Ca^2+^ crosslinking, post-printing treatment was crucial. As a result, they obtained an outstanding printability of hSF-laden bioink, which made possible 3D bioprint complex structures with a precise cell density and well-defined porosity. Furthermore, these 3D-printed scaffolds exhibited shape and size stability and cell viability for around one month. The bioactive features coupled with excellent printability properties make this alternative hybrid bioink an attractive biomaterial for skin tissue engineering, wound healing, and drug testing platform.

### 3.4. Hyaluronic Acid

Hyaluronic acid (HA) is a natural heteropolysaccharide from the glycosaminoglycans groups (GAGs) [180], that was first isolated from bovine eyes by Meyer and Palmer in 1934 [181]. As well as other GAGs, HA is composed of repeating disaccharide building blocks consisting of a uronic sugar (β−1,4-D-glucuronic acid) and an amino sugar (β−1,3-*N*-acetyl glucosamine) [182]. However, HA differs from other GAGs as it is not sulfated; it is synthesised by hyaluronan synthases and it can have a wide range of molecular weights, depending on the source [183,184]. Under physiological conditions, HA exists in the form of the negatively charged hyaluronate macromolecule and its corresponding salts. This polyanionic hyaluronan is highly hydrophilic since it interacts with water a thousand times more than the neutral polymer, improving its combination with different intra and extracellular tissues components [185].

HA is one of the most important constituents of the extracellular matrix (ECM) and due to its capability to retain water in the ECM, it plays a key role in filling organ spaces (vitreous humor and skin), absorbing shock impacts (cartilage), and lubricating moving tissues (joints) [186]. In addition to contributing to the structure and physiological properties of connective tissues and body fluids, HA participates in various biological processes, such as morphogenesis, inflammation, tissue restoration and regeneration, homeostasis, maintenance of ECM integrity, and mediation of cellular functions [187]. Furthermore, HA acts as a signaling molecule controlling cell adhesion, migration, and proliferation [188].

Due to its favourable features, such as biocompatibility, biodegradability, bioresorbability, high viscosity, and mechanical stability, HA is an ideal biomaterial for designing and developing non-adhesive, non-thrombogenic, and non-immunogenic scaffolds for tissue engineering and wound dressing purposes [189,190,191]. For this reason, HA has been extensively used as bioink for 3D printing for materials fabrication with biomedical applications [192,193,194]. To be employed as a 3D printable bioink, HA requires being chemically modified and mixed with other polymers to improve the rheological and mechanical properties. In a recent work, Hauptstein et al. studied different printable bioink compositions based on HA to achieve homogeneous ECM distribution for engineered constructs with biological properties [195]. For this, thiolated HA and allyl-modified poly(glycidol) were UV-crosslinked and supplemented with a 1 wt% unmodified high-molecular weight HA (HWHA) to adapt bioink to polycaprolactone(PCL)-supported 3D bioprinting. As a result, using an extrusion-based printing process, they obtained gels with a low concentration of polymers (3 wt%) and supplemented them with HWHA, showing an enhanced stiffness and homogeneous ECM distribution in 3D bioprinted, PCL-supported scaffolds. The multifunction of this HA-based bioink supplement, that both allows PCL-supported bioprinting and increases the quality of the developing 3D scaffolds, is promising for many applications in biofabrication.

Another group developed an alternative bioprinting gel by combining HA with hydroxyethyl acrylate (HEA) and gelatin-methacryloyl (GM), HA-g-pHEA-GM, to be used as a bioink in tissue engineering [196]. In this study, bioink synthesis consisted in a first graft polymerisation of HA and HEA and then a second grafting of GM via radical polymerisation mechanism. After that, the bioink printing ability was evaluated by using a home-built, multi-material 3D bioprinting system with pneumatic and piston extrusion. HA-based hydrogel demonstrated excellent properties such as good swelling, printability, morphology, biocompatibility, stable rheology, and drug delivery capabilities. This study proved that the HA-g-pHEA-GM hydrogel can be successfully 3D printed and has a strong potential to be used as a bioink for tissue regeneration applications. Closely related to this, Lee et al. also used acrylated HA to develop a dual function hybrid bioink with a short gelation time and biological functions [197]. To achieve mechanical integrity and fast gelation time, HA was conjugated with tyramine (HA-tyr) and mixed with acrylated HA in a ratio of 1:9 to achieve a storage modulus G’ of 1 kPa that enables higher cell proliferative activity. Once the hybrid hydrogel was obtained, they tested the printability of the viscous bioink using a lab-made 3D microextrusion bioprinter and evaluated the stem cell viability after printing. They observed that printed hydrogels conserved their mechanical properties and preserved the viability of incorporated stem cells. As well as this, an optimised HA-tyr bioink was obtained by a mechanism of two consecutive crosslinking steps comprising a first enzymatic crosslinking reaction mediated by horseradish peroxidase (HRP) and hydrogen peroxide (H_2_O_2_), followed by a green light crosslinking triggered by Eosin Y photosensitiser [198]. For cell-laden bioinks, fibroblasts, chondrocytes, or MSCs were added before the enzymatic crosslinking step, after which the printing process starts. Combining different concentrations of HRP and H_2_O_2_, viscoelastic properties of the new HA-tyr bioink were easily tunable, achieving a soft bioink that could be extruded through a thin needle. Finally, by exposing the bioink at 505 nm during the printing procedure, 3D constructs carrying viable cells were obtained. Due to their simplicity and versatility, these novel bioinks based on HA-tyr can be exploited for biofabrication of a wide variety of tissue-engineered constructs using an ECM component combined with different cell types.

### 3.5. Alginic Acid

Alginic acid salt, commonly known as alginate, is one of the most popular and abundant biopolymers available in nature [199]. It is derived from the cell wall of brown seaweed, and from the capsule of some micro-organisms, such as Azotobacter sp. and Pseudomonas sp. Alginate is an anionic polysaccharide composed of linear copolymers including (1,4)-linked β-D-mannuronic (M) and (1,4)-α-L-guluronic (G) acid units that are arranged in M-blocks, G-blocks, and in heteropolymeric sequences of alternating M and G residues. The sequence and ratio of G and M, as well as the molecular weight (32,000 to 400,000 g/mol) of alginate depend on the type of the natural source. Purified alginates have the capability to generate hydrogels by the crosslinking of carboxylate groups of G residues with divalent cations (Ca^2+^, Ba^2+^, Sr^2+^, and Mg^2+^). Thus, alginic acids with a high G concentration tend to form stiffer hydrogels, while alginates with low G content yield softer elastic materials [200,201].

The similar structure of alginate to the extracellular matrix coupled with its biocompatibility, nontoxicity, biodegradability, low cost of extraction, and ease of gelation processes, make alginate-based hydrogels ideal candidates in the design and fabrication of bioinks [145,202], for several biomedical applications, such as wound healing, regenerating human tissues, drug delivery, and cell culture [203,204,205]. For example, in 2019, Wang and cowokers printed alginate directly into viscous pre-polymers of hydrogels including gelatin methacrylate, agarose, and gelatin to form microchannels for the creation of a vascular network for drug screening, tissue engineering, and organ-on-a-chip [206]. As well as this, Freeman et al. study how the mechanical properties of 3D printed constructs can be tuned by changing the molecular weight of alginate bioinks, gelling conditions, and choice of ionic crosslinker [207]. Besides, they discovered that by modulating the stiffness of 3D bioprinted, alginate-based hydrogels, mesenchymal stem cell differentiation can be regulated and, hence, complex tissues can be engineered.

Despite alginic acid being a frequently used bioink in 3D bioprinting, due to its poor stability and soft mechanical properties, alginate is commonly combined with other materials, like distinct natural or synthetic polymers, to form new composites with improved characteristics. As an example, for increasing viscosity, methylcellulose or gelatin are usually added to alginate to enhance printability and degradation kinetics [208]. For instance, Luo et al. mixed CNF with gelatin–alginate thermal-responsive bioinks to improve the bioprinting properties of the hydrogels [209]. They prepared six different hydrogels with varying contents of gelatin and CNF, and examined their printability by a home-made microextrusion bioprinter. Mechanical properties were evaluated before and after crosslinking with CaCl_2_, and viability and metabolic activity of cells entrapped in the bioprinted structures were also tested. As a result, they found that bioinks composed of 20% (*w/v*) gelatin, 1.25% (*w/v*) alginate, and 0.25% (*w/v*) CNF presented better distribution of cells and an increased viscosity compared to the hydrogels without CNF, indicating that the combination of the three components are crucial to obtain a scaffold with superior printability and higher biocompatibility. In another study, calcium alginate was mixed with agar to prepare a new bioink with improved printing resolution and to enhance the mechanical properties of the 3D bioprinted structures [210]. Agar had the function of increasing the viscosity of the ink and thus its rheological properties, while alginate connected different layers by crosslinking with Ca^2+^ to give a better interface in the 3D printed hydrogels. Furthermore, by introducing a soft polyacrylamide network into the 3D printed, alginate-based hydrogels, interfacial defects were minimised, obtaining 3D constructs with outstanding mechanical properties, high biocompatibility, shape fidelity, and high permeability. Chitosan was also used as an enhancer of the rheological properties of alginate bioinks. As chitosan is insoluble in aqueous solutions, Liu et al. proposed to incorporate chitosan powders into the alginate solution to make a 3D printing ink with superior viscosity [211]. After printing the bioink by a 3D-BIOPLOTTERTM, a hydrochloric acid (HCl) solution was added to the deposited fibres to solubilise the chitosan and enable the formation of alginate–chitosan hydrogels. As a result, they observed that physicochemical properties of the alginate-based bioink could be manipulated by modifying the concentration of chitosan, and the obtained 3D printed hydrogels provided an appropriate environment for cell growth and differentiation. This strategy not only allows the use of 3D printing to develop neo tissues or organs, but it also to repairs damaged ones. In a similar approach, the incorporation of carboxylated cellulose nanocrystals and/or xanthan gum in sodium alginate hydrogel inks provided improved post-printing fidelity, and rheological and mechanical properties. Furthermore, good viability of the human skin fibroblast was observed highlighting the potentialities of the developed 3D bioprintable hydrogel inks [212]. More recently, 3D printed dressings composed of gelatin methacrylate and xanthan gum with the incorporation of the antimicrobial *N*-halamine and TiO_2_ nanoparticles were reported. The incorporation of the *N*-halamine provided a wide-spectrum of antimicrobial activity, while the nanoparticles improved the ultraviolet stability of *N*-halamines. This three-dimensional antibacterial wound dressing presented good antibacterial activity, outstanding biocompatibility, and significantly accelerated the wound healing in a mouse model [213].

### 3.6. Other Biopolymers

Apart from polymers exploited from natural resources, synthetic biopolymers are also used as bioinks for 3D bioprinting. Synthetic polymers are manmade polymers usually obtained by chemical reactions with tunable chemical and physical features [214]. Compared to natural biopolymers, synthetics have superior mechanical properties and have a crucial role in conserving cellular and biomolecular functions before, during, and after the 3D printing procedures. They can be easily modified for improving physicochemical properties, and also functionalised with different molecules to meet particular requirements [215]. Among the synthetic polymers that are commonly printed are polylactic acid (PLA) [216], polyethylene glycol (PEG) [217], polycaprolactone (PCL) [218], polyglycolic acid (PGA) [219], polyurethane (PU) [220], and polylactic-co-glycolic acid (PLGA) [221].

The proper mechanical properties of synthetic bioinks are advantageous to withstand the stresses suffered during 3D printing stages and in vivo implantations. Furthermore, synthetic polymers have controllable degradation kinetics and are easy to process, light weight, non-toxic, inexpensive, and abundant, which might be convenient when choosing a material to print scaffolds for biomedical applications. Although they can be successfully used as bioinks for 3D printing in their pure form, synthetic polymers are also combined with reinforcing materials to develop mechanically superior structures with optimised regenerative action and higher printability [222]. However, it is of paramount importance to study the fate and effect of nanomaterials [223,224,225]. Indeed, a recent report evaluated the use of reinforcement materials (carbon nanotubes, copper, and steel). Using a condensation particle counter, it was possible to measure 10^5^–10^6^ particles emissions per cm^3^ from these materials. Furthermore, the authors provide important insights about cellular metabolic alterations, intracellular mitochondrial stress, and toxicity as a result of particle emissions [226].

It is worth mentioning that both natural and synthetic polymers have been simultaneously printed for the fabrication of advanced scaffolds for tissue engineering [227,228,229].

## 4. Applications of 3D Printed Biopolymers for Skin Wound Healing

### 4.1. Role of Bioinks on Skin Bioprinting

The 3D bioprinting technique represents a promising alternative approach to produce scaffolds that can be employed as a personalised therapeutic method to accelerate wound healing and protect against infections. In this sense, different scaffolds combining biopolymers, nano-objects, cells, and therapeutic molecules have been successfully reported. For example, it was possible to facilitate the extrusion printing process of gelatin-methacryloyl-based bioink with the addition of an ulvan type polysaccharide. The 3D bioprinted, cell-laden scaffolds support cell viability and proliferation of human dermal fibroblasts [230]. Alternatively, tyrosinase was employed to crosslink gelatin methacrylamide and collagen bioinks, and consequently improve their mechanical strength. The enzyme also plays an important role in the skin regeneration process [157]. Furthermore, a novel PLA scaffold combined with chitosan and loaded with Cu-carbon dots, rosmarinic acid, and hyaluronic acid was produced employing a 3D bioprinting method. This complex, bioprinted structure includes antimicrobial agents (i.e.: Cu-carbon dots, rosmarinic acid, chitosan), biocompatible polymers (i.e., PLA and chitosan), and a natural polymer existing in skin (hyaluronic acid). The resulting bionanocomposite scaffolds possess antimicrobial activity and non-toxicity, and significantly increase the expression of genes involved in wound healing (i.e., GAP, PDGF, TGF-β, and MMP-1), and improve wound healing properties in vivo [231]. Similarly, an antibacterial bioink based on alginate and methylcellulose, loaded with Ga^+3^, was developed. In this case, the Ga^+3^ ions contribute to stabilise the bioink through the formation of ionic crosslinks with alginate, and the resulting material possesses potent antimicrobial activity against both Gram positive and Gram negative bacteria [143].

**Figure 13:**
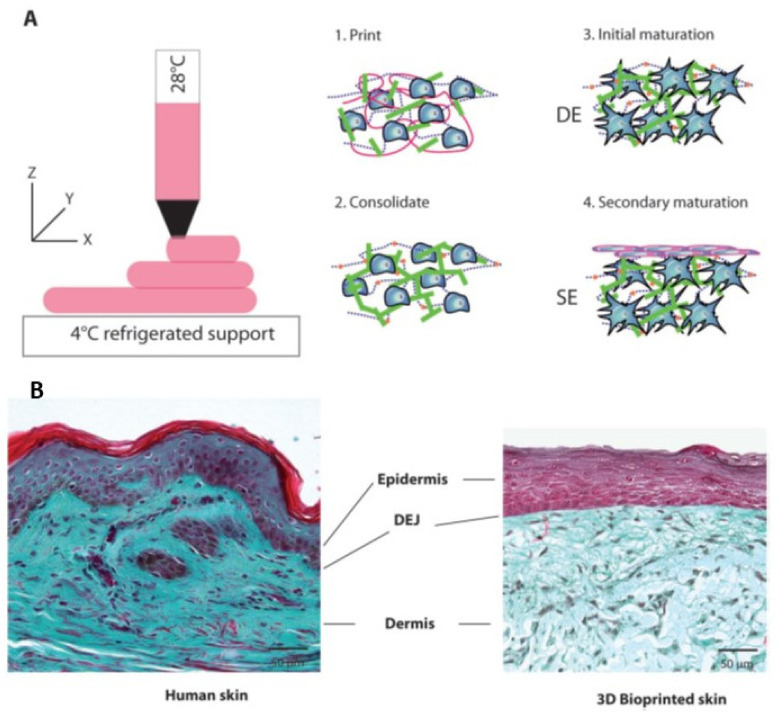
(**A**) Schematic representation of the 3D bioprinting, consolidation, and maturation steps using the developed bioink. (**B**) Histological and morphological characterisation of the bioprinted skin. Optical microscopy images of normal human skin and bioprinted skin after 26 d of culturing. Tissues were stained with Masson’s Trichrome. Reproduced [232] with permission from John Wiley and Sons, 2016.

**Figure 14:**
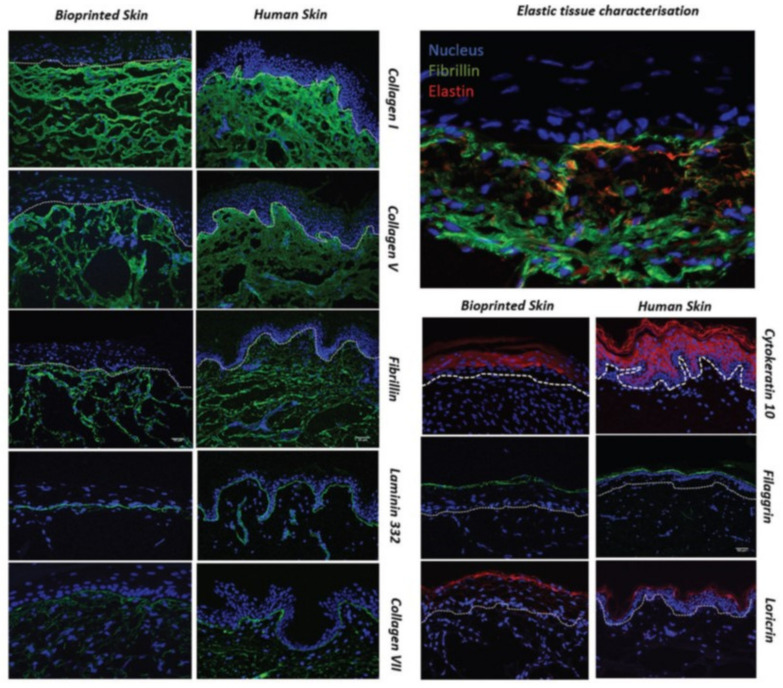
Epidermal differentiation and dermal markers’ profiles of bioprinted skin in comparison to normal human skin from a healthy donor. Fluorescent microscopy observations. Reproduced from [232] with permission from John Wiley and Sons, 2016.

A skin model mimicking the dermis and the epidermis with its cellular, molecular, and macromolecular features was produced using a bioink formulation composed of a mixture of gelatin, alginate, and fibrinogen [232]. An in-house-built, open-source machine was used for the 3D printing of a 5 mm-thick artificial dermis with extension in the centimetre range by an extrusion process in a matter of minutes. Each bioink component had a role on the skin bioprinting. Gelatin offered appropriate rheology during the extrusion process, strength when the formulation is printed on a cooled substrate, and solubility for being eliminated in subsequent steps. Alginate gave structural stiffness and stability once the gelation was eliminated, owing to its calcium-based hydrogel formation. Fibrinogen, on its side, offered structural stability by the crosslinking with alginate and promoted cellular maturation based on the presence of RGD domains. Bioprinted dermis was achieved by printing objects composed of primary human dermal fibroblasts immersed in the bioink formulation with a subsequent culture. Primary human epidermal keratinocytes were then seeded on top of the bioprinted dermis for the generation of the bioprinted skin (Figure 13A). Although in contrast to normal dermis, the bioprinted dermis only contained fibroblasts, cellular morphology, viability, and organisation, and epidermal proliferation and differentiation in the bioprinted skin resembled that of normal human skin (Figure 13B). The expression of several epidermal markers (Ki67, cytokeratin 10, filaggrin, and loricrin), extracellular matrix proteins (collagen I and V, vimentin, fibrillin, and elastin), and laminin 332 at the dermal–epidermal interface supported these observations (Figure 14). Ultrastructural analysis of the bioprinted skin revealed the presence of corneodesmosomes in the stratum corneum, keratohyalin granules in the stratum granulosum, several desmosomes in the stratum spinosum, and many hemidesmosomes linked to keratin filaments in the basement membrane and mature collagen fibres. The 3D bioprinting capability of the reported process was also evaluated by producing an adult-sized ear by printing the fibroblasts containing bioink, which retained the organisation after the culture [232].

It is still a challenge to develop biocompatible bioinks with rapid gelation kinetics and tunable mechanical properties. A bioink suitable for rapid printing of bio-inspired 3D tissue constructs has been recently reported. The bioink was composed of gelatin methacrylate (GelMA), *N*-(2-aminoethyl)-4-(4-(hydroxymethyl)-2-methoxy-5-nitrosophenoxy) butanamide (NB), linked hyaluronic acid (HA-NB), and photo-initiator lithium phenyl-2,4,6 trimethylbenzoylphosphinate (LAP). Interestingly, after UV irradiation, the hydrogel was rapidly formed at t ≈ 1.384 s, while without the addition of LAP, the gel was formed at t ≈ 33 s. A significantly higher compressive modulus was achieved with this formulation when compared to single crosslinking hydrogels. Moreover, it was possible to prepare (c.a. 3 min.) a dense upper layer and a porous lower layer mimicking the epidermal layer and corium layer, respectively. The hydrogel possesses remarkable biocompatibility with cell viability rates superior to 95%, provoking limited inflammation after subcutaneous implantation and facilitating wound healing in vivo [233].

### 4.2. Role of Cell Seeding

Alternatively, seeding and cultivating cells in 3D printed scaffolds is becoming an active field of research. Especially, 3D printing technology allows the introduction of multiple cell types within specific positions in the scaffolds and the survival rates would be really high [3,5]. Indeed, adult human dermal fibroblasts and adult human epidermal keratinocytes can survive and grow after being 3D bioprinted with a hydrogel scaffold [234]. In addition, the efficacy in full-thickness burn wound healing in a rat model of a 3D bioprinted collagen and alginate scaffold was reported. The material was arranged layer-by-layer with and without the addition of adipose-derived mesenchymal stem cells. The burn wound healing results of employing cellularised materials were far more effective than acellularised treatments [235]. Meanwhile, it was reported that an autologous homologous adipose tissue, prepared employing 3D bioprinting, successfully accelerated diabetic wound healing with complete wound closure and re-epithelialisation within four weeks [236]. Interestingly, Zhang et al., developed a skin model with sweat glands and hair follicles. According to the authors, the difficulties in simultaneously inducing sweat glands and hair follicle regeneration have been overcome with this model [237]. In parallel, gelatine-sodium alginate hydrogel loaded with adipose-derived mesenchymal stem cells was constructed by 3D bioprinting. The incorporation of a NO donor, such as S-Nitroso-N-acetyl-D, or L-penicillamine, successfully protects against ischaemia and reperfusion injury, and improves the proangiogenic potential of the cells. Indeed, the bioprinted scaffold effectively promotes wound healing in a severe burn model [238].

Another biomimetic skin model which was qualitatively and quantitatively characterised was constructed by a 3D-printing-assisted electrohydrodynamic jetting process [239]. The construct was composed of an acellular polycaprolactone/collagen scaffold that served as a resting layer for consecutive fibroblast/collagen and keratinocyte/collagen layers. The extrusion process of the cellular bioinks was previously simulated and modelled in order to find the experimental conditions that preserved cellular viability and function. The bioprinted model was qualitatively and quantitatively compared with manually seeded skin equivalents. Metabolic activity and cell viability assays in both skin equivalents revealed the positive effect of the fibroblast layer on keratinocyte layer. Moreover, keratinocyte differentiation and the formation of orthokeratinised epidermis were evidenced and the morphology of full-thickness skins was similar to normal human skin. Immunohistochemical analysis showed the specific localisation of differentiation markers: vimentin in the dermal layer and keratin K14 y K10 in the lower and upper layers of the epidermis, respectively. Moreover, keratin K2 was colocalised with filaggrin in the upper layers, which is associated with skin barrier function. Laminin V and collagen IV showed a robust presence in the dermal–epidermal junction. Occludin, E-cadherin, and plakoglobin were also evidenced in both skin equivalents, demonstrating intact organisations and architectures. Skin barrier function assays also revealed similar results for 3D printed and manually seeded models. Stress (keratin 16), water channel activity (aquaporin 3), DNA damage (γ-H2AX), and oxidative stress (catalase) markers showed similar patterns to those observed in normal human skin. However, both skin constructs did not show normal full differentiation after two weeks in the culture, and further improvement of the 3D bioprinting methodology would be needed.

### 4.3. Role of Incorporated Therapeutic Agents

The advances in this field have led to the development of biocompatible scaffolds with incorporated specific cell types, but a next step would be the generation of interconnected functional vessels to mimic the sophisticated architectural and biological structure of the skin [240]. In this sense, the incorporation in the bioinks of therapeutic agents with the ability to stimulate blood vessel formation is highly desirable. Silica-based materials are known to stimulate collagen deposition and blood vessel formation during the wound healing process [148,241,242]. Similarly, Sr ions can stimulate the expression of angiogenic factors in cells and, thus, promote the angiogenesis [243,244].

**Figure 15:**
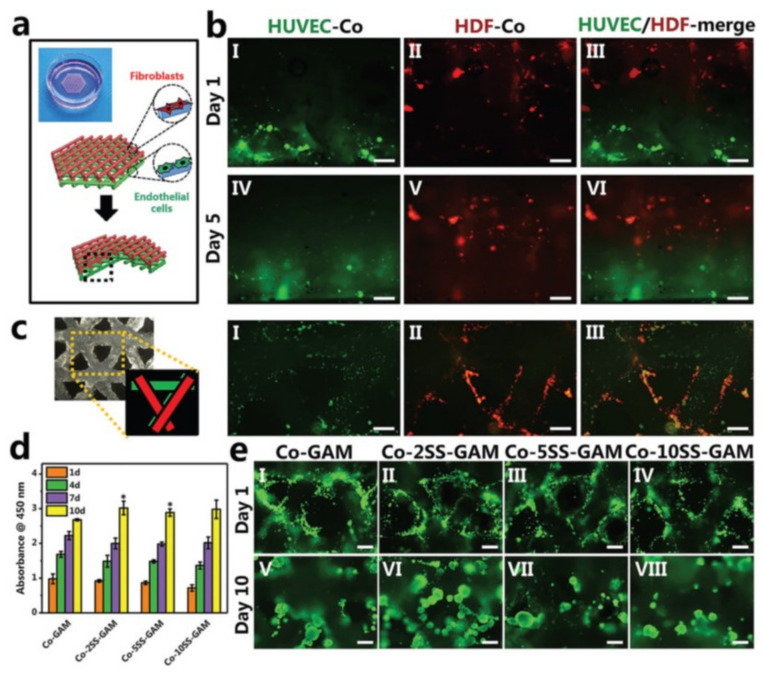
Spatial distribution and proliferation activity of cells in 3D bioprinting Co-SS-GAM biomimetic multicellular scaffolds. (**a**) Schematic illustration of the spatial distribution of HDFs and HUVECs in the Co-2SS-GAM scaffold. (**b**,**c**) Fluorescence images of the printed cells on the (**b**) vertical section and (**c**) horizontal direction of the scaffolds. HUVECs were labeled with green cell trackers and HDFs were labeled with red cell trackers. After culturing for one and five days, the microscope photographs of (I,IV) HUVECs, (II,V) HDFs, and (III,VI) HUVEC/HDF merged in the same area showed the bilayer distribution of two kinds of cells. Scale bar: 150 μm. (**d**) Proliferation behaviour of co-cultured cells in the Co-GAM, Co-2SS-GAM, Co-5SS-GAM, and Co-10SS-GAM scaffolds for 1, 4, 7, and 10 days (*n* = 3, * *p* < 0.05). (**e**) Live/dead assay of the co-cultured cells in the scaffolds on day 1 (I to IV) and day 10 (V to VIII). Scale bar: 150 μm. Reproduced from [245] with permission from John Wiley and Sons, 2021.

**Figure 16:**
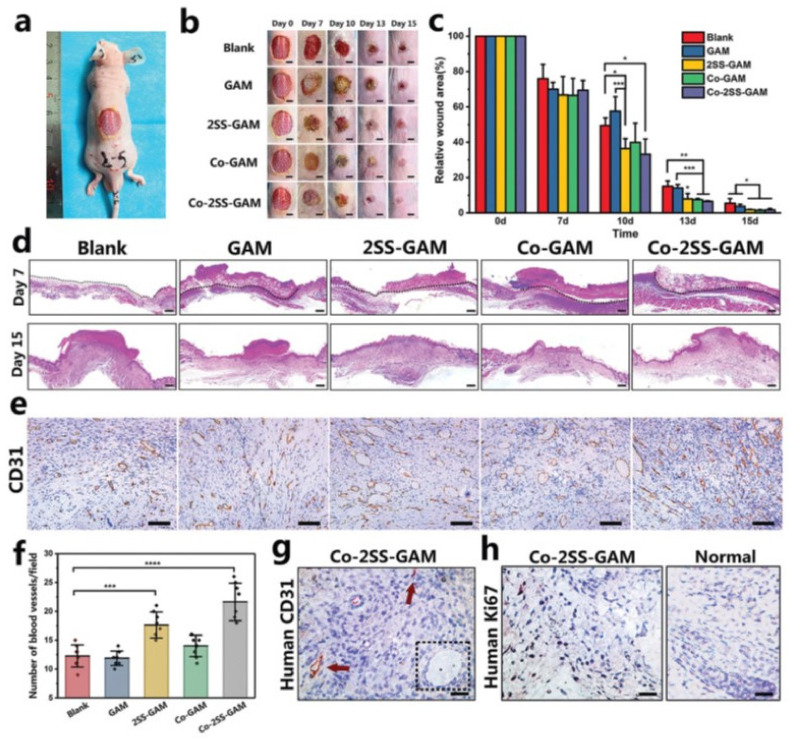
Induction of skin tissue regeneration in nude mice. (**a**) Visual appearance of nude mice after graft of 3D bioprinting scaffolds onto full-thickness skin defects. (**b**,**c**) Gross photos of murine skin wounds and statistics of wound closure rates of the blank, GAM (cell-free), 2SS-GAM (cell-free), Co-GAM (cell-laden), and Co-2SS-GAM (cell-laden) groups on days 0, 7, 10, 13, and 15 (*n* = 4). Scale bar: 5 mm. (**d**) H&E staining of sections of skin tissue obtained from all groups on day 7 and day 15. The black dotted line marked the boundary between the wound and scaffolds on day 7. Scale bar: 500 μm. (**e**) Images of CD31 immunohistochemical staining exhibited denser blood vessels in SS-containing groups (2SS-GAM and Co-2SS-GAM) than in the other groups. The Co-2SS-GAM group showed the highest degree of angiogenesis. Scale bar: 100 μm. (**f**) Quantification of blood vessels in the regenerative dermis on day 15 (*n* = 8). (**g**) Immunohistochemical staining of specific human CD31 confirmed a few human blood vessels (red arrows) formed by the transplanted HUVECs in Co-2SS-GAM group. The black dotted frame marked the host blood vessel. Scale bar: 30 μm. (**h**) The activity of the printed cells in Co-2SS-GAM group was shown by human Ki67 antibody staining (brown: human Ki67 and blue: mouse nucleus). Scale bar: 30 μm. * *p* < 0.05, ** *p* < 0.01, *** *p* < 0.001, **** *p* < 0.0001. Reproduced from [245] with permission from John Wiley and Sons, 2021.

With the conviction that the recapitulation of dermal vasculature is an essential step for the generation of optimal bioprinted skin substitutes, strontium silicate microcylinders were integrated in a bioink to achieve an enhanced vascularisation [245]. High crystalline microparticles with a diameter of 15 µm were synthesised by a hydrothermal method and showed a continuous release pattern of strontium and silicon ions, which have proven to stimulate collagen deposition and angiogenesis during wound healing. The strontium silicate microcylinders were incorporated in a bioink composed of a mixture of gellan gum (GAM), sodium alginate, and methyl cellulose, with good flexibility and printability. The preparation of the biomimetic skin scaffolds included the air-pressure-induced extrusion of the microcylinder-doped bioink and the overlaid spraying of human umbilical vascular endothelial cells or human dermal fibroblasts using a piezoelectric pipette. This was performed in a cyclical manner reaching layer-by-layer structures of bioink and cell suspensions. In turn, the two cell types were included in two different major layers, with human umbilical vascular endothelial cells in the bottom and human dermal fibroblasts in the top, in order to emulate the vascularised native skin structure (Figure 15). The gene expression of several angiogenic markers, such as vascular endothelial cadherin, endothelial nitric oxide synthase, vascular endothelial growth factor, and hypoxia-inducible factor-1*α*, were detected in the printed cells and showed higher levels than in the same cell-seeded bioprinted scaffolds. In view of these results, the in vivo vascularisation and skin regeneration in acute and chronic wounds of the prepared biomimetic skin substitutes were tested. When the multicellular scaffolds were subcutaneously implanted in nude mice, a large number of blood vessels with a CD31 (endothelial cell junction marker) protein expression and an enhanced collagen I deposition were found. In an acute wound mouse model, a completed epithelialisation and dermal structure recovery with enhanced angiogenesis and active proliferation of regenerated skin was observed in the transplanted animals after 15 days (Figure 16). Meanwhile, a diabetic mouse model was used to study the level of skin regeneration induced by the grafted, bioprinted skin substitutes. The results showed a high healing rate with cells on wound beds that actively recovered and had a rebuilt dermis vasculature, demonstrating a prominent repair of complex skin chronic wounds.

### 4.4. Full-Thickness Functional Skin Models

Full-thickness skin wounds are physiologically complex and require biomaterials that mimic the inherently sophisticated structure and function of the dermis. For this purpose, researchers designed and printed a bilayer membrane scaffold consisting of: (i) an outer poly (lactic-co-glycolic acid) (PLGA) membrane which maintained the moisture content of the hydrogel and prevented bacterial invasion, and (ii) a lower alginate hydrogel layer which promoted cell adhesion and proliferation in vitro. This structure was designed to mimic the skin epidermis and dermis. This scaffold successfully improved collagen I/III deposition, neovascularisation, and skin regeneration [246].

**Figure 17:**
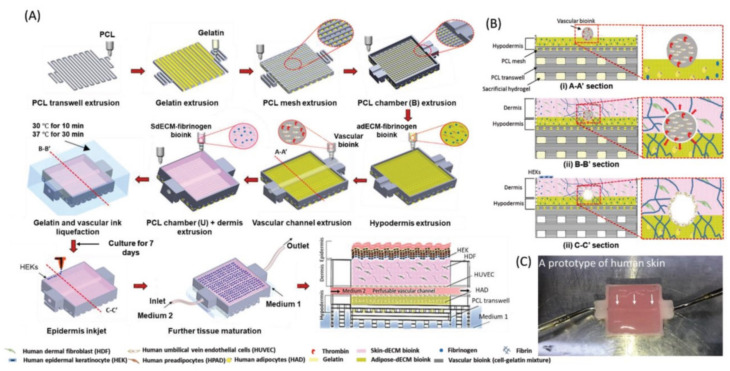
The 3D cell printing process for fabrication of 3D P/V full-thickness skin model. (**A**) Schematic diagram exhibiting the step-by-step fabrication process. (**B**) Sectional views provided from the aforementioned fabrication process. (**C**) A prototype of the fabricated skin construct. Reproduced from [249] with permission from John Wiley and Sons, 2018.

**Figure 18:**
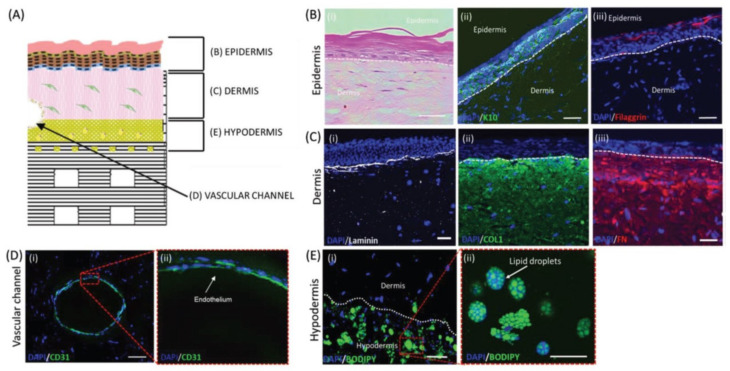
Histological analyses representing skin tissue maturation in in vitro environment. (**A**) Illustration of each zone of epidermis, dermis, hypodermis, and vascular channel. (**B**) Epidermis stratified (H&E staining) and stained with keratin 10 (K10) and filaggrin representing early differentiation and late differentiation of epidermis, respectively. (**C**) Dermis imaged with protein markers representing epidermal–dermal junction (Laminin) and secreted ECM components (COL1: collagen type I and FN: fibronectin). (**D**) Vascular channel in the mature 3D human skin equivalent stained with CD31 demonstrating the presence of endothelial cells. (**E**) Hypodermis stained with BODIPY representing lipid droplets of adipocytes (Scale bars: 50 μm). Reproduced from [249] with permission from John Wiley and Sons, 2018.

Jin et al. developed a full-thickness functional skin model. The bioprinted scaffold is formed by gelatin methacrylamide with HaCaTs cells as an epidermal layer, acellular dermal matrix with fibroblasts as the dermis, and gelatin methacrylamide mesh with HUVECs cells as the vascular network and framework. This bioprinted skin model stimulates dermal extracellular matrix secretion and angiogenesis, promotes wound healing and re-epithelisation, and, overall, improves wound healing quality [247]. Tuener et al., reported a promising strategy to produce prevascularised regenerative scaffolds for wound care. For this purpose, a bioink containing a core of a peptide-functionalised, succinylated chitosan and dextran aldehyde, cell-laden material was covered by a shell of gelatin methacryloyl. Two cell types were delivered with the bioink: (i) in the core of HUVECs and (ii) in the shell of hBMSCs. Wound closure with this system was increased two-fold [248]. In an effort to improve the structural complexity of bioprinted skin and produce a model more similar to the native human skin, a perfusable and vascularised structure composed of epidermis, dermis, and hypodermis strati was achieved [249]. This model was 3D printed following several steps of fabrication (Figure 17). First, a transwell based on polycaprolatone was extruded to reach a 15 × 15 × 6 mm structure, followed by the extrusion of a sacrificial gelatin hydrogel for the filling of the construct pores. A hypodermal compartment 2 mm high was constructed on top by the generation of a microporous polycaprolatone mesh and the extrusion of a preadipocytes-embedded, adipose-fibrinogen bioink. Afterwards, a bioink containing human umbilical vein endothelial cells and thrombin-embedded gelatin hydrogel was printed as cylindrical vascular channels. The gelatin component allows the cylinder shape to endure during fabrication as well as further liquefaction at 37 °C, leaving perfusable hollow channels and promoting the attachment of the human umbilical vein endothelial cells to the surface of the generated channel. A dermal compartment of 3 mm high was then printed using a bioink composed of human dermal, fibroblast-encapsulated skin–fibrinogen. The crosslinking of the fibrinogen component was triggered when the vascular bioink containing thrombin was liquefied. Two different culture media were used for the maturation of the skin structure and could be infused, owing to the model design: fibroblasts growth/preadipocyte differentiation medium and endothelial growth medium. Primary human epidermal keratinocytes were deposited onto the dermal stratum by injecting 3D cell printing. The porous transwell and the generated vascular channel were used to infuse proper media, to promote differentiation and maturation of the different cellular components and the final conformation of the skin construct, including lipid droplet-associated hypodermis, extracellular matrix-secreted dermis, and stratified epidermis. Functional markers in the structures, which are characteristic of each layer (i.e., the stratified structure of the epidermis, the dermal–epidermal junction, and extracellular matrix of the dermis, as well as the lipid droplets of the hypodermis, and the endothelium of the vascular channels), were evaluated (Figure 18). The maturation of the epidermic layer was demonstrated by the expression of keratin 10 and filaggrin at early and late stages of cellular differentiation. The epidermal–dermal junction formation was revealed by the expression of laminin, collagen type I, and fibronectin in the interface between layers. The lipid droplets in mature adipocytes were exposed by their staining with boron-dipyrromethene in the hypodermis. Vascular channels showed full coverage with human umbilical vein endothelial cells, as revealed by the expression of the CD31 marker. Further epidermal compartment evaluation was realised in comparison with skin models including only the dermis and the epidermis, and with native skin. The expression of the p63 stemness marker and the K19 follicular stem cell marker demonstrated that the full-thickness skin model had epidermal stratification and hypodermis/epidermis crosstalk, similar to the native skin. However, the ki67 proliferation marker expression revealed a possible non-sufficient provision of nutrients and oxygen through the straight bioprinted vascular channel. Although this perfusable platform would be useful for incorporating other cells for advanced biomimetic skin models, the hypodermis in this study was thinner than that corresponding in the native skin. This could limit the substantial influence of the hypodermis.

Another multicellular and multilayer biomimetic skin structure was achieved [250] by a 3D printing process using gelatin methacryloyl and alginate-based bioinks. The 3D skin substitute was composed of three main compartments. The bottom one was prepared by extrusion of a bioink containing gelatin methacryloyl and alginate, and including human umbilical vein endothelial cells on a polyester porous membrane, in order to guarantee the access of media. The mixture of gelatin methacryloyl and alginate allowed for obtaining a bioink with enhanced gelation, printability, and rheological properties, and for maintaining the viability of printed cells. The middle compartment was generated by pouring a gelatin methacryloyl matrix containing human dermal fibroblasts, followed by a UV-crosslinking process. The stiffness of the matrix was adjusted to guarantee the growth and function of the dermal cells. In fact, these were revealed by the positive staining of Ki-67 and F-actin, high levels of Pro-Collagen I alpha 1, and low levels of Matrix Metalloproteinase I. The top compartment was achieved by seeding multiple layers of human epidermal keratinocytes with a gelatin coating to achieve a c.a. 200 µm-thick biomimetic epidermis. The entire skin structure showed a good organisation of layers with no mixture within cell layers. However, the angiogenic activity of the human umbilical vein endothelial cells, as well as the differentiation of human epidermal keratinocytes and the formation of the stratum corneum have not yet been assessed using this skin model.

### 4.5. The 3D Bioprinted Alternative Skin Models

The 3D bioprinting technology allows the production of intricate structures with desired patterns, biological activities, and physiological functions, providing a unique approach for the fabrication of artificial tissues. Particularly, the combination of precise cell deposition, reproducibility, high yields, versatility, and high efficiency of 3D bioprinting offers the opportunity to reproduce the complex human skin heterogeneity. Engineered skin not only provides advanced constructs to better replicate human skin, but also agrees with policies that tend to reduce the use of laboratory animals as in vivo models [251,252]. In agreement with 3R principles (replacement, reduction, and refinement) defined by Russell and Burch in 1959 for animal use in research [253], the current regulations and ethical concerns on animal testing and the need of skin substitutes with more physiological functions explain the significant advances in the development of 3D, innovative skin models in the last years [254]. For example, at present, the European Union has decreed the prohibition of the use of animals for testing of cosmetic ingredients. In this context, the French cosmetics company L’Oreal has partnered with the US-based bioprinting firm, Organovo, to develop 3D bioprinted human skin for the testing of their products without using people or animals.

The 3D human-based cell cultures in vitro have many advantages over the use of animal models. For example, variables are better controlled than in the case of in vivo complex organisms, enhancing reproducibility and simplifying the research of cellular and molecular processes. Furthermore, as bioprinted human skin models contain human cells, they can better mimic the in vivo environment, replicate cell morphology and adhesion, and promote cell differentiation, proliferation, and migration, providing an accurate platform to obtain more predictable results for humans [255]. This also offers the possibility to develop a personalised medicine through the use of autologous patients’ cells or tissues, avoiding the risk of immunological rejection that is very common when animal tissues are transplanted into humans [256].

Madiedo-Podvrsan et al., reported the first human-patterned epidermal model created by the use of a high-precision 3D bioprinting approach [257]. In order to mimic pathological human skin and improve research into damaged skin without using animal models, they bioprinted separate populations of keratinocytes with normal or low filaggrin expressions in a single model insert, to reproduce healthy skin and human epidermal disorders, respectively. This technique has the potential to create a heterogeneous and stable reconstructed model of two skin conditions in a single sample, better reflecting native human skin, reducing results variability, and avoiding the use of a large number of animal models in vivo that often do not accurately predict human responses.

Another alternative bioprinting approach used to replicate complex papillary dermis structures and reduce the gap between in vitro and in vivo models was reported by Park et al [258]. They designed a technique to fabricate self-organised 3D collagen microstructures through inkjet fibroblasts bioprinting. By using drop-on-demand inkjet printing, they could seed a controlled number of fibroblast cells in aligned patterns onto a collagen substrate prepared by microextrusion printing. The formation of a vertically elevated collagen-based 3D microstructure was obtained after cells interacted and rearranged the surrounding extracellular matrix. Finally, they inkjet-printed human keratinocytes onto the fibroblast-mediated, 3D-protruded collagen microstructures to fabricate a bilayered skin model that could mimic the papillary interface at the dermo–epidermal junction. As a result, they obtained 3D microstructures containing fibroblasts that were covered by the printed human keratinocytes. This approach to create 3D cell-laden collagen microstructures offers an innovative way to reproduce the structure and functions of human skin, making an important contribution to replace animal testing and shorten the distance between in vitro and in vivo skin models.

Due to the limited control of three-dimensional structures and contraction of engineered materials achieved by current protocols, Derr et al. developed another bioprinting method for the fabrication of skin equivalents (SEs) with comparable morphology and functions of native skin tissue, providing a new platform for improving wound healing therapies, transplants for regenerative medicine, and testing of skin products [259]. The Ses were fully bioprinted on an open-market printer in a layer-by-layer model, in a multiwell-based platform. The material structure consisted in three levels: the dermis, containing neonatal human dermal fibroblasts; laminin/entactin basal layer; and the epidermis loaded with neonatal normal human epithelial keratinocytes. The constructs were validated by immunohistochemistry, impedance measurement, permeation assays, and cell viability assays, showing a viable material with optimal barrier function and reproducibility that allowed their used as tissue models for diseases screening. The production of SEs in an automatised and standardised manner was also approached by Cubo et al [255]. In this case, they used a free-form fabrication 3D bioprinting technique to engineer a human plasma-derived bilayer skin using human fibroblasts and keratinocytes. The most innovative result of this method was the capability to reproducibly print large areas of human skin, which is imperative to improve the actual treatments of different skin pathologies such as burns, ulcers, and surgical wounds.

Although 3D bioprinted skin models appear as an attractive substitute for animal use, most of them have certain drawbacks, such as the absence of immunologic components. In this sense, several attempts have been made to improve 3D models by incorporating immunogenic components, such as immune cells, to obtain new immunocompetent, three-dimensional materials with human immune system features that will benefit the treatment of infections, the study of inflammatory pathologies, and the development of novel therapies for other skin diseases [260]. For example, BASF Care Creations^®^ and CTIBiotech laboratories recently developed the first 3D bioprinted skin models, including immune macrophages, to reconstruct skin tissues for the development and testing of bio-actives for advanced skin care applications [261]. This technology provides a new system with more human physiological properties that will allow the study of the activity of macrophages in a complete reconstructed skin. Furthermore, as macrophages are essential for wound healing, tissue regeneration, and inflammation control, this novel immunocompetency will improve the research, development, and evaluation of skin care products. To reproduce the protective skin functions, Poblete Jara et al. also developed a 3D bioprinted human skin equivalent with immune responses by including macrophages, keratinocytes, and fibroblasts in a collagen matrix [262]. For this, they first created a bilayer skin model by the extrusion of a bioink composed of collagen I and primary fibroblasts, followed by the extrusion of the keratinocyte solution on top of the fibroblast–collagen layer. After 11 days, they induced a wound in the centre of the skin model and printed a fibrin clot-macrophages bioink, and the healing process was evaluated from 0 to 10 days. As a result, they observed that the SE containing macrophages showed a complete re-epithelisation after 10 days post-wounding, compared to the SE without the macrophage bioink treatment, which revealed an incomplete wound closure. Furthermore, they contrasted these results with a murine dermal wound model and observed that in both cases, a new layer of keranocytes was formed in the wound centre, indicating that the 3D human skin platform can replace animal models and guarantee comparable results.

Besides the lack of immune components, 3D bioprinted skin models often lack vascularisation, which is also essential for the graft take. The group of Baltazar et al. produced by 3D printing a vascularised SE through the incorporation of human foreskin dermal fibroblasts and endothelial and placental cells to form a dermis, and human foreskin keratinocytes to construct an epidermis [263]. For this, the dermal and epidermal layers were printed in two steps. Firstly, the vascularised dermis was bioprinted with endothelial cells and fibroblasts and cultured for four days to stimulate vascularisation; secondly, the epidermis was bioprinted with keranocytes on day four and cultured in a skin differentiation medium. As well as human skin, the bioprinted skin models showed positive Ki67 and CK14 expressions in the epidermis, indicating a regular keranocytes proliferation. They also found that by allowing endothelial cells to self-assemble into vessels, a complex structure similar to natural tissues is formed. In fact, it was crucial to have an in vitro maturation time to obtain SE with an equivalent human skin arrangement. This shows that despite 3D bioprinted skin still being in its early stages and requiring improved of many factors, it provides a new potent alternative for developing human skin replicates for tissue engineering.

### 4.6. Four-Dimmensional Printing

Stimuli-responsive materials represent an emerging type of materials employed for wound healing. Recently, Municoy et al. summarised that a variety of stimuli such as magnet fields, temperature, redox-state, pH, and light were employed to change a material’s structure, dimensions, and properties for tissue engineering and drug delivery [264]. In a step forward, stimuli-responsive materials have been employed with 3D bioprinting technology in the so-called 4D bioprinting, where printed objects change their structure or properties with time, when an external stimulus is applied [265]. Different 4D bioprinting strategies would be employed to produce these 4D bioprinted structures that undergo shape or functional transformations over time [266]. This disruptive technology, which allows printing responsive materials that can change their shape, or materials that can reorganise with cellular self-organization, has broadened the applications of 4D bioprinting in various biomedical fields, such as tissue engineering and drug delivery [267,268,269].

The principle of dynamic movement was recently achieved employing hydroxybutyl methacrylated chitosan as a temperature-responsive polymer. The expansion of the 4D structure was provoked by the expansion of water at a low temperature. On contrary, when the temperature rose, deswelling and, consequently, a decrease in the volume, occurred [270]. Biomedical 4D scaffolds were developed with renewable plant oils (soybean oil-epoxidised acrylate). The material fixed a temporary shape at a low temperature (−18 °C) and at 37 °C it fully recovers the original shape. In addition, it supports cell addition and proliferation, which confirms the great potential for biomedical applications [271]. Similarly, 4D printed hierarchy scaffolds with high biocompatibility, a microporous structure, and tunable shape recovery speed for tissue engineering applications were reported [272].

A multifunctional ionic skin was fabricated by the 3D printing of a thermo-responsive hydrogel (composed of n-octadecyl acrylate and poly-dimethylacrilamide) into a capacitor circuit [273] for the monitoring of body temperature, finger touch, and bending motion. The proposed hydrogel exhibited elastic activity with a volume phase transition temperature around 30 °C, and it is ionically conductive in the presence of salt solutes. The hydrogel’s viscosity decrease through heating allowed its 3D printing using an ink extrusion system. A skin-like capacity sensor was constructed by surrounding a dielectric polyethylene layer with two grid-structure hydrogel films with a sub-millimeter resolution. These films exhibited an enhanced capacitive response compared to the bulk hydrogel with the temperature increasing, which depends on the film’s area. The fabricated sensor offered wearability and looked transparent when deposited onto human skin. The capacitive response was sensitive to temperature and compressive pressure changes with a reversible behaviour. When the sensor was subjected to changes in both the temperature and the pressure, the sensor did not show a linear response towards these parameters in a wide range, which, according to the authors, could be improved by tunning the structure of the sensor.

## 5. Conclusions

The increasing demand for tissue engineering scaffolds cannot be achieved by traditional technologies such as natural scaffolds or tissue donors. In this sense, a combination of materials enhances the properties such as biocompatibility, biodegradability, tensile strength, and design development for the additional cell seeding. In this sense, Liang et al., recently reported recent developments in advanced, functional hydrogel dressings with outstanding properties such as antioxidant, anti-inflammatory, antimicrobial, therapeutic delivery, self-healing, stimuli-response, conductivity, and wound monitoring properties [274]. In parallel, Guo et al. highlighted the advantages and limitations of haemostatic materials that aid wound healing [275]. This is an important step in the wound healing process and a great advance involving natural and synthetic polymers, silicon-based materials; and metal-containing materials in the form of particles, fibres, sponges, and hydrogels have been reported. Even though, the growing world needs a break-through in scaffold fabrication techniques. In this vein, 3D printing technology is a promising tool due to its versatility and capacity to offer different synthesis strategies with a wide variety of materials and their combinations. It is important to combine traditional knowledge with the current new technologies and give rise to multifunctional developments.

3D bioprinting technologies have enormous potential in tissue engineering, regenerative medicine, and drug development. The last few years have seen how 3D bioprinting technologies have evolved and become more sophisticated to fabricate specific human organs and tissues such as skin. However, as the goals for printing more complex tissues progress, new challenges arise, including bioprinting of soft materials, printing resolution, and speed and reproducibility of the printing process to develop high-throughput 3D bioprinting. Another important field of research is the development of bioinks with suitable properties and characteristics of the desired tissue.

The lack of self-availability and the time between scaffold bioprinting and their use raises various concerns. In this sense, a recent work described the possibility to print and, at the same time, freeze, the biomaterials. This crybioprinting method developed by Zhang and col. allows the direct fabrication and in situ freezing of tissue constructs, maintaining the functionality of the cells and making them shelf available [276].

The speed of 3D printing is highly influenced by the complexity of the structure and the number of required voxels since most of the 3D printers produce materials point-by-point or layer-by-layer. To overcome this issue, Yang et al. employed wavelength-sensitive photoresins which can be cured simultaneously by employing visible and UV sources in a tomographic volumetric printing process to offer fast 3D printing [277].

Finally, since the bioprinting process has a lot of complexities, a future goal could be the application of machine learning (ML) and a computational method collection, which contains mathematical functions of the real world based on historical data. In this sense, ML could overcome the complexity of representing biological tissue models from tissue images into a 3D tissue model with cellular resolution and tissue properties, and the compatibility of different materials used could be predicted [278,279]. In addition, the combination of ML with Big Data, related to modern clinical images, could help solve the multiscale and multiparameter complexities when the number of changing parameters is exceeded in the processing and post-processing process. In this vein, Big Data sources for 3D bioprinting could be the different diagnostic images, experimental data, and the scientific literature [280]. As a concluding remark, 3D bioprinting is a promising tool in tissue engineering that could be improved with the addition of ML.

## Figures and Tables

**Table 1:** Characteristics, advantages and 3D printing technologies used with collagen, chitosan, cellulose, hyaluronic acid, and alginate-based bioinks.

Bioink	Characteristics	Advantages	3D Printing Technology	Examples of 3D Techniques in Literature	Examples of Cell-Laden Three Dimensional (3D) Bioprinting
Collagen-based bioink	Natural polymer material, good biocompatibility, promotes cell adhesion, proliferation, and migration. It is safe for the host and does not cause serious inflammation. It is enzymatically degradable [133].	High porosity, absorbability, low immunogenicity [134]. High cell adhesion [135].	It can be printed at low temperatures and forms a solidified gel at body temperature [133]. At low concentrations (0.1 wt%), collagen is suitable for droplet ejection, inkjet, and laser-assisted 3D bioprinting. At higher concentrations (above 1.25 wt%), it reaches a viscosity suitable for extrusion [136].	MVB, EB, IBP, DOD, LBP [11].	Human primary foreskin-derived dermal fibroblasts [137]
Chitosan-based bioink	Chitosan is derived from chitin, a polysaccharide from the exoskeleton of shrimp and other sea crustaceans. It has a linear structure, which can be quickly formed into a gel matrix using NaOH [133].	Chitosan has good biocompatibility and biodegradability [133]. Mild gelation conditions and antibacterial properties [134].	Chitosan-based hydrogels are usually used with an extrusion bioprinter and there are a low number of studies of chitosan printed by jet-based bioprinting methods [138].	EB [11].	Keratinocyte and human dermal fibroblast cells [139]
Cellulose-based bioink	Cellulose is a linear polysaccharide, the most abundant natural polymer in nature. It is biocompatible and nontoxic [140].	The cellulose hydroxyl groups are available for chemical modification by esterification, graft copolymerisation, etherification, selective oxidation, or intermolecular crosslinking reaction, leading to vast possibilities in bioink formulation [141].	It is used in bioinks as reinforcing material with good bio-adhesion and mechanical properties [140].	EB [142]	Fibroblasts [143]
Hyaluronic acid-based bioink	Hyaluronic acid is an anionic polysaccharide that promotes tissue regeneration. Low molecular weight hyaluronic acid can promote cell differentiation and angiogenesis [133].	Excellent moisture retention and promotes cell proliferation [134].	It can be used alone, but it is more commonly used in combination with other biomaterials to improve the physical properties of the bioink mixture [136].	EB, PEI [11].	Human dermal fibroblast [144]
Alginic acid-based bioink	Low cell adhesion [135]. Alginate is a naturally derived linear polymer from the cell wall of brown algae. Alginate is a polysaccharide that is negatively charged. This soluble biopolymer supports cell growth and exhibits high biocompatibility [145].	Easy, fast gelation and low cost [134].	Many bioinks described in the literature are composed of alginate or in combination with other biopolymers. The popularity can be explained by the simplicity of the ionotropic gelation process, and because of the network precursor, sodium alginate, which is commercially available and cheap [136].	EB, LIFT, MVB [11].	Human amniotic epithelial cells and Wharton’s jelly-derived mesenchymal stem cells [146]
